# Association of the C‐Reactive Protein–Triglyceride–Glucose Index With All‐Cause and Cardiovascular Mortality in U.S. Adults: Insights From NHANES 1999–2010

**DOI:** 10.1155/cdr/4209201

**Published:** 2026-10-08

**Authors:** Xin Wang, Siyuan Ma, Xinrui Hai, Ali Ma, Peng Wu, Xiaolan Liang, Yu Hao, Libo Yang, Ning Yan

**Affiliations:** ^1^ First Clinical College Ningxia Medical University Yinchuan Ningxia China nxmu.edu.cn; ^2^ Heart Centre & Department of Cardiovascular Diseases General Hospital of Ningxia Medical University Yinchuan Ningxia China nxmu.edu.cn; ^3^ Department of Cardio-Cerebro-Pulmonary Diseases Lingwu Hospital of Traditional Chinese Medicine Lingwu Ningxia China; ^4^ Institute of Medical Sciences General Hospital of Ningxia Medical University Yinchuan Ningxia China nxmu.edu.cn

**Keywords:** all-cause mortality, cardiovascular disease, C-reactive protein-triglyceride-glucose index, NHANES

## Abstract

**Background:**

CTI combines C‐reactive protein with the triglyceride–glucose index and may capture concurrent inflammatory and insulin–resistance‐related metabolic disturbance. Emerging cohort evidence has linked higher CTI values to all‐cause and CVD mortality, but the robustness, dose–response characteristics, and predictive relevance of these associations remain uncertain. This study evaluated the associations of CTI with all‐cause and CVD mortality among U.S. adults.

**Methods:**

Participants were selected from NHANES survey cycles conducted from 1999 through 2010, yielding an analytic sample of 10,528 adults aged 18 years or older. The CTI was computed using the formula: CTI = 0.412 × ln [CRP (mg/L)] + ln {[triglycerides (mg/dL) × fasting glucose (mg/dL)]/2}. We used weighted Cox proportional hazards models to investigate the relationships of CTI with all‐cause and CVD mortality. Restricted cubic spline analyses were used to assess nonlinearity, followed by exploratory two‐piecewise Cox regression to examine possible changes in slope. Beyond the primary analysis, the robustness and potential heterogeneity of these associations were further assessed using sensitivity, subgroup, and interaction analyses.

**Results:**

During a survey‐weighted mean follow‐up of 164.29 ± 50.47 months, the primary survey‐weighted analytic sample included 10,528 participants. In the primary DAG‐informed Cox models, each 1‐unit increase in CTI was associated with higher risks of all‐cause mortality (HR = 1.24, 95% CI: 1.18–1.31, *p* < 0.001) and CVD mortality (csHR = 1.35, 95% CI: 1.20–1.53, *p* < 0.001). Compared with Q1, participants in Q4 had higher risks of all‐cause mortality (HR = 1.54, 95% CI: 1.29–1.84, *p* < 0.001) and CVD mortality (csHR = 1.78, 95% CI: 1.27–2.49, *p* < 0.001). These associations remained significant after additional clinical adjustment. Exploratory two‐piecewise analysis estimated an inflection point of 8.165 for all‐cause mortality (conventional likelihood‐ratio *p* = 0.002); because the breakpoint was selected using a data‐driven procedure, this comparison was interpreted cautiously. For CVD mortality, the estimated inflection point was 8.070 and the two‐piecewise model did not clearly improve fit over the linear model (*p* = 0.067). Time‐dependent AUCs for CTI were approximately 0.60–0.62 at 5, 10, and 15 years, indicating modest discrimination.

**Conclusion:**

After adjustment for prespecified covariates, higher CTI was associated with greater hazards of all‐cause and CVD mortality. Exploratory analyses suggested a possible change in slope in the association with all‐cause mortality, whereas evidence for a distinct change point in cardiovascular mortality was inconclusive. These findings extend recent population‐based evidence linking CTI to mortality; however, its standalone discrimination and incremental predictive improvement beyond established clinical risk factors were modest. CTI may therefore be better viewed as an adjunctive marker of inflammatory‐metabolic burden than as a major addition to existing clinical prediction models.

## 1. Introduction

Cardiovascular disease (CVD) remains one of the major contributors to premature mortality and adverse health outcomes worldwide [1–4], resulting in approximately 17.9 million deaths each year, which is nearly one‐third of worldwide fatalities [5]. The modern global health landscape is increasingly defined by a compounding crisis: As populations age rapidly and metabolic disorders like diabetes proliferate, CVD continues to represent a major burden on public health systems, contributing substantially to morbidity and mortality through consistently high prevalence and fatal outcomes [6, 7]. Metabolic disorders, particularly Type 2 diabetes, frequently coexist with overt CVD. Together, these factors contribute to a higher burden of adverse cardiovascular events and greater complexity in disease management, resulting in substantially worse long‐term outcomes [8–11]. Despite considerable advances in medical interventions and risk factor management, the prevalence of CVD remains a challenge for healthcare systems globally [12, 13]. The early identification of important factors linked to CVD development, progression, and mortality is essential for enhancing preventative and management strategies.

Insulin resistance (IR) is a metabolic condition characterized by diminished responsiveness to insulin, resulting in impaired glucose uptake by peripheral tissues. A growing body of evidence suggests that IR is strongly associated with the progression of atherosclerotic disease [14–16]. IR commonly accompanies chronic hyperglycemia, dyslipidemia, obesity, and additional metabolic irregularities [17]. IR has been reported to contribute to vascular endothelial dysfunction through inflammatory activation, oxidative stress, and increased platelet adhesion and aggregation. These mechanisms may promote thrombosis, particularly in individuals with hypertension [18]. Moreover, IR‐induced metabolic disturbances can accelerate atherosclerotic development [14, 15]. Although the hyperinsulinemic‐euglycemic clamp is considered the gold standard for IR assessment, its complexity and expense make it unsuitable for large‐scale population research [19]. Against this backdrop, alternative markers based on conventional biochemical biomarkers have attracted widespread attention. Among these biomarkers, the triglyceride‐glucose index (TyG) has attracted substantial attention. Calculated using fasting triglyceride and glucose levels, TyG was first proposed in 2008 and is now commonly considered a reliable proxy for IR. Earlier studies have reported good agreement between TyG and established reference methods, such as the hyperinsulinemic‐euglycemic clamp, while also supporting its usefulness in predicting CVD risk across different population groups [20, 21]. This measure has therefore been widely used in epidemiological investigations, clinical research, and risk stratification because of its simplicity and clinical relevance [22, 23]. Substantial evidence derived from longitudinal cohorts and pooled analyses firmly establishes TyG and its derivative indices as consistent predictors of a broad range of cardiometabolic conditions, including diabetes, stroke, heart failure, coronary artery disease, and atherosclerosis [24–26].

Persistent low‐grade inflammatory activity is considered central to the pathogenesis of a broad range of cardiovascular and metabolic conditions. It may contribute to the development and rupture of atherosclerotic plaques, thereby facilitating thrombus formation and increasing the risk of myocardial infarction, stroke, and other major cardiovascular events [27]. In addition, inflammatory activity has been strongly linked to unfavorable cardiac changes, such as ventricular remodeling and impaired myocardial function [28, 29]. CRP is a widely utilized biomarker of inflammation. Increased concentrations of CRP, a marker of the inflammatory response, have been shown to both indicate the presence of inflammation and predict CVD risk [30]. Accumulating data suggest a relationship between elevated CRP concentrations and heightened CVD risk, implying that CRP could serve as an adjunctive biomarker for cardiovascular risk evaluation and early high‐risk screening [31]. However, the CRP marker alone is insufficient for a full characterization of systemic inflammatory activity, which is well documented in research as a primary contributor to the advancement of atherosclerosis [32]. IR and inflammation may interact bidirectionally: IR contributes to inflammatory pathway activation through lipotoxicity and oxidative stress, whereas inflammation may further disrupt insulin signaling [33]. Therefore, a composite biomarker integrating IR and systemic inflammation may improve the assessment of cardiometabolic risk, offering a novel approach to categorizing diverse groups with varying metabolic profiles. Ruan et al. first proposed CTI [34], with the proposal entailing the linear combination of CRP with the TyG index. This approach was adopted to achieve a unified quantification of IR and systemic inflammation. CTI has demonstrated prognostic value for cancer mortality [35]. Previous research has demonstrated a close association between CTI and NAFLD [36]. In addition to CTI, several inflammatory biomarkers, including interleukin‐6 (IL‐6), tumor necrosis factor‐alpha (TNF‐*α*), NLRP3 inflammasome components, calprotectin, neopterin, lactoferrin, and neutrophil extracellular traps (NETs), have been linked to an increased risk of CVD, potentially through inflammatory and immune‐mediated pathways [37–43]. Prior evidence suggests that CTI is a useful prognostic indicator in individuals with hypertension, cerebrovascular diseases, and selected cancers. However, few population‐based studies have investigated whether CTI is associated with all‐cause and CVD mortality in the general adult population [35, 44].

Previous NHANES‐based studies have already reported associations between CTI and mortality in U.S. adults. Accordingly, the present study was not designed to establish this association de novo, but rather to examine its robustness, functional form, and potential clinical relevance using a broader analytic framework. Specifically, we incorporated fasting‐subsample survey weighting, a prespecified DAG‐informed adjustment strategy, analyses addressing selection related to complete‐case inclusion, proportional‐hazards diagnostics, competing‐risk methods, exploratory assessment of nonlinearity and possible changes in slope, and evaluation of incremental predictive performance. Using NHANES 1999–2010, we therefore examined the associations of CTI with all‐cause and CVD mortality across these complementary analytic approaches.

## 2. Methods

### 2.1. Data Source and Study Population

Data were extracted from NHANES 1999–2010, a National Center for Health Statistics survey designed to provide nationally representative information on the health and nutritional status of noninstitutionalized residents of the United States. The study protocol followed NHANES ethical requirements, and written informed consent was provided by every participant. In addition, the study design and reporting adhered to the STROBE statement to support transparency and rigor. The study initially included 62,160 individuals from NHANES 1999–2010. After restricting the study population to adults aged ≥ 18 years, 35,379 participants were eligible for further assessment. Exclusions were then applied sequentially so that each participant was counted only once at the first applicable exclusion step. We excluded 3865 participants with missing CRP measurements, 16,213 with missing triglyceride measurements among the remaining participants, and 40 with missing fasting glucose measurements among those remaining. We subsequently excluded 16 participants with missing mortality status, followed by 505 with missing marital status, 1211 with missing poverty‐income ratio (PIR), 918 with missing smoking status, 851 with missing alcohol consumption information, and 492 who were pregnant at the time of examination. After these exclusions, 11,268 participants remained eligible. Because CTI incorporates fasting triglycerides (TG) and fasting glucose, analyses incorporating NHANES survey weights required the fasting subsample weight. Therefore, an additional 740 participants without a valid positive fasting subsample weight were excluded, yielding a final survey‐weighted analytic sample of 10,528 participants. Potential selection related to participant inclusion was examined through baseline comparisons by CTI availability and an inverse‐probability‐of‐selection weighted sensitivity analysis. The participant selection process is shown in Figure 1.

**Figure 1:**
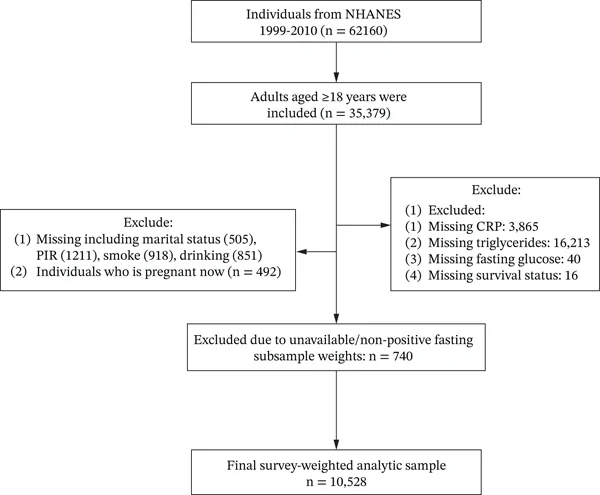
Flowchart of the sample selection from NHANES 1999–2010.

### 2.2. Measurement of C‐Reactive Protein (CRP)–Triglyceride–Glucose Index

Fasting blood samples were collected during the morning examination sessions and processed according to standardized NHANES laboratory procedures. CRP, TG, and fasting plasma glucose (FPG) were measured according to cycle‐specific NHANES laboratory protocols. Briefly, CRP was measured by nephelometry, TG were measured using enzymatic methods, and FPG was measured using hexokinase‐based enzymatic methods. Because laboratory instruments and analytic platforms varied across NHANES survey cycles, detailed assay procedures and instrumentation for each cycle are available in the corresponding NHANES laboratory documentation. Fasting laboratory parameters, including CRP, TG, and FPG, were used to calculate CTI. For CTI calculation, CRP concentrations were converted from mg/dL to mg/L, whereas triglyceride and FPG concentrations were expressed in mg/dL. CTI was calculated using the following formula:

CTI=0.412×ln CRPmg/L+lntriglyceridesmg/dL×fasting glucosemg/dL2.

CTI was initially evaluated as a continuous measure and then divided into quartiles for supplementary analyses.

### 2.3. Assessments of Other Covariates

Trained personnel gathered baseline information following standardized procedures, comprising household interviews, physical evaluations at mobile examination centers (MECs), and laboratory analyses. Covariates included sociodemographic factors, lifestyle factors, anthropometric measurements, medical history, medication use, and laboratory parameters. Sociodemographic and lifestyle variables included age, gender, race/ethnicity, marital status, PIR, educational attainment, smoking status, and alcohol consumption. Clinical variables included hypertension, diabetes, dyslipidemia, chronic kidney disease (CKD), CVD, weight and body mass index (BMI), blood pressure, urine albumin‐to‐creatinine ratio (UACR), and CRP, FPG, glycated hemoglobin (HbA1c), total cholesterol (TC), TG, high‐density lipoprotein cholesterol (HDL‐C), low‐density lipoprotein cholesterol (LDL‐C), hemoglobin (HGB), estimated glomerular filtration rate (eGFR), serum creatinine (SCR), and relevant medication use. CTI and other derived variables were calculated from these laboratory and clinical indicators. Covariate selection for the primary multivariable model was guided by a directed acyclic graph (DAG) representing the hypothesized causal relationships between CTI, potential confounders, and mortality outcomes. Under the causal assumptions represented in the DAG, the minimally sufficient adjustment set comprised age, gender, race/ethnicity, educational attainment, marital status, PIR, smoking status, and alcohol consumption.

### 2.4. Definitions

Participants were classified as hypertensive when they fulfilled at least one of the following criteria: (1) a recorded systolic blood pressure ≥ 140 mmHg, (2) measured diastolic blood pressure of 90 mmHg or higher, (3) self‐reported diagnosis of hypertension by a physician, or (4) currently using antihypertensive medication. Diabetes mellitus (DM) was defined as meeting any of the following criteria: (1) FPG ≥ 126 mg/dL, (2) HbA1c levels at or above 6.5%, (3) regular use of diabetes medications, (4) a formal diagnosis confirmed by a medical practitioner. Prediabetes was defined as FPG levels falling between 100 and 125 mg/dL, whereas HbA1c hovers in the 5.7%–6.4% range. In contrast, normal glucose regulation (NGR) is characterized by FPG under 100 mg/dL combined with HbA1c readings below 5.7%. Abnormal cholesterol levels were defined as TG ≥ 150 mg/dL, TC ≥ 240 mg/dL, HDL cholesterol < 40 mg/dL, LDL cholesterol ≥ 160 mg/dL, current use of lipid‐lowering statins, or a physician‐diagnosed history of dyslipidemia. These criteria were consistent with commonly used clinical guidelines for identifying dyslipidemia [45].

### 2.5. Mortality Data in the NHANES Cohort

Mortality status and underlying cause of death were ascertained from the National Center for Health Statistics 2019 Public‐Use Linked Mortality Files, which link eligible NHANES participants to the National Death Index, with follow‐up through December 31, 2019. All‐cause mortality was defined using the final mortality status variable (MORTSTAT), with MORTSTAT = 1 indicating death from any cause. CVD mortality was defined as death due to diseases of the heart or cerebrovascular diseases, corresponding to UCOD_LEADING = 001 or 005, respectively. These categories correspond to ICD‐10 codes I00–I09, I11, I13, I20–I51 for diseases of the heart and I60–I69 for cerebrovascular diseases. Follow‐up time was defined using PERMTH_INT, representing the number of person‐months from the NHANES interview date to death or the end of mortality follow‐up.

### 2.6. Statistical Analysis

Unless otherwise specified, survey‐weighted descriptive analyses and association models accounted for the complex, multistage probability sampling design of NHANES using sampling weights, strata, and primary sampling units (PSUs). Because CTI required fasting triglyceride and FPG measurements from the morning fasting subsample, fasting subsample weights were used. Following NCHS guidelines for combining NHANES 1999–2010 data, a 12‐year fasting subsample weight (WTSAF12YR) was constructed as WTSAF4YR/3 for 1999–2002 and WTSAF2YR/6 for the 2003–2004, 2005–2006, 2007–2008, and 2009–2010 cycles. The survey design specified SDMVSTRA as the stratum, SDMVPSU as the PSU, and WTSAF12YR as the sampling weight; variances were estimated by Taylor series linearization. Participants without a valid positive fasting subsample weight were excluded. Complete‐case analysis was used for multivariable Cox models, with participants missing model‐specific covariates excluded only from the corresponding adjusted model. To evaluate potential selection associated with model‐specific covariate completeness, an inverse‐probability‐of‐selection weighted sensitivity analysis was performed. The probability of retention in the complete‐case analysis was estimated using logistic regression including age, age squared, gender, race/ethnicity, educational attainment, and NHANES survey cycle. Stabilized inverse‐probability‐of‐selection weights were calculated and multiplied by the NHANES fasting‐subsample survey weights to obtain combined analysis weights, while retaining the original NHANES strata and PSUs. Weight stability was assessed using the distribution of the stabilized selection weights and the Kish effective sample size calculated from these weights among participants included in the final IPW mortality analyses. Baseline characteristics were summarized by CTI quartiles as survey‐weighted means ± standard deviations for continuous variables and unweighted counts with survey‐weighted percentages for categorical variables. Conventional unweighted methods were used for Kaplan–Meier/log‐rank analyses, proportional‐hazards diagnostics, exploratory threshold analyses, standalone time‐dependent ROC analyses, and incremental prediction analyses, as specified below.

Survey‐weighted Cox regression was used to estimate HRs and 95% CIs for CTI in relation to all‐cause and CVD mortality. Model 1 was unadjusted. Model 2, the primary DAG‐informed model, adjusted for age, gender, race/ethnicity, educational attainment, marital status, PIR, smoking status, and alcohol consumption. Model 3 additionally adjusted for hypertension, CKD, and HGB. CRP, TG, and FPG were not entered separately because they are components of CTI. BMI, diabetes, hyperlipidemia, and TC were evaluated in sensitivity analyses because of their close metabolic relationships with CTI, and eGFR was evaluated as an alternative to CKD; SCR was not included as a separate covariate. For CVD mortality, primary analyses used cause‐specific Cox models, with deaths from causes other than CVD treated as censored observations; estimates are therefore reported as cause‐specific hazard ratios (csHRs). Survey‐weighted Fine–Gray models were additionally fitted as competing‐risk sensitivity analyses, with non‐CVD death treated as the competing event. Fine–Gray interval weights were combined with the NHANES fasting‐subsample weights while retaining the original strata and PSUs; results are reported as subdistribution HRs (sHRs) with 95% CIs. The hypothesized causal structure and primary adjustment set are illustrated in Figure S1.

Multicollinearity was assessed using generalized variance inflation factors (GVIFs), with GVIF^(1/(2 × Df))^ reported for variables with more than one degree of freedom. The proportional hazards (PH) assumption was assessed using scaled Schoenfeld residuals in conventional unweighted Cox models specified with the same covariates as the corresponding survey‐weighted models. Because these diagnostics were not obtained directly from the complex‐survey Cox estimator, they were treated as model‐diagnostic sensitivity analyses. When evidence of nonproportionality was identified, additional models incorporating stratification and/or time‐varying terms were fitted to evaluate the stability of the CTI effect estimates; detailed diagnostics and corrected model specifications are reported in Table S6 and Figures S2, S3, S4, and S5. Multicollinearity results and correlation analyses are reported in Tables S3 and S4, respectively.

Tests for trend assigned each CTI quartile its median value and modeled these values continuously, whereas spline models were used to examine departures from linearity with the same covariate adjustment as the extended clinical model. When suggested by the spline analysis, exploratory two‐piecewise Cox regression was performed using conventional unweighted Cox models in the same complete‐case sample. Breakpoints were obtained using the original data‐driven likelihood‐based search, and the linear and two‐piecewise models were compared using a conventional likelihood‐ratio test. Because breakpoint selection was data driven, the likelihood‐ratio comparison was considered exploratory rather than confirmatory; confidence intervals for the selected change points were not calculated.

Kaplan–Meier curves and log‐rank tests were used to compare survival across CTI quartiles. For CVD mortality, deaths from non‐CVD causes were treated as censored observations in the Kaplan–Meier analysis; accordingly, these curves represent cause‐specific survival rather than cumulative incidence. Time‐dependent discrimination of CTI, CRP, and TyG was evaluated at 5, 10, and 15 years using cumulative/dynamic AUCs estimated by inverse probability of censoring weighting as implemented in the timeROC package. These standalone timeROC analyses were conducted using conventional unweighted methods. The censoring distribution was estimated using the Kaplan–Meier method (weighting = “marginal”); these IPCW weights were used to account for censoring and were not NHANES survey sampling weights. Pointwise 95% CIs and pairwise AUC comparisons were obtained using the iid‐representation‐based procedures implemented in timeROC. For CVD mortality, non‐CVD mortality was treated as a competing event.

To assess the incremental predictive value of CTI beyond established clinical risk factors, a separate prediction‐oriented conventional risk‐factor model was constructed including age, gender, race/ethnicity, smoking status, hypertension, DM, BMI, TC, HDL cholesterol, and CKD. This model was compared with an otherwise identical model additionally including continuous CTI, with both models fitted in the same complete‐case prediction sample. Prediction analyses were conducted using conventional unweighted methods. For all‐cause mortality, discrimination was assessed using Harrell′s C‐index. For CVD mortality, discrimination was assessed in a competing‐risk framework using time‐dependent AUCs at 5, 10, and 15 years, with non‐CVD death treated as a competing event. Prediction error was evaluated using Brier scores at the corresponding time points with inverse‐probability‐of‐censoring weighting; the censoring distribution was estimated using the Kaplan–Meier method. Calibration was assessed in deciles of predicted risk. Observed all‐cause mortality risk was estimated using local Kaplan–Meier methods, whereas observed CVD risk was estimated using local Aalen–Johansen methods with non‐CVD death treated as a competing event. Decision curve analyses were performed at the same time horizons using model‐predicted risks, with treat‐all and treat‐none strategies shown as reference strategies. All prediction metrics were evaluated in the same complete‐case sample used for model fitting and therefore represent apparent performance; no cross‐validation or optimism‐corrected internal validation was performed. The 95% confidence interval for the change in Harrell′s C‐index was estimated using 2000 paired bootstrap resamples, with both models refitted within each bootstrap sample; this bootstrap was used for uncertainty estimation of the apparent C‐index difference rather than for optimism correction. Confidence intervals for differences in time‐dependent AUCs and Brier scores were obtained using influence–function‐based inference. Subgroup and interaction analyses were considered exploratory because multiple interactions were examined without adjustment for multiplicity.

## 3. Results

### 3.1. Baseline Characteristics of Participants and Outcome Parameters

The primary survey‐weighted analytic sample included 10,528 participants, with a survey‐weighted mean follow‐up time of 164.29 ± 50.47 months. Baseline characteristics are shown in Table 1. Participants were progressively older across increasing CTI quartiles (weighted mean age: 39.86 ± 15.06 years in Q1 and 50.87 ± 15.70 years in Q4; *p* < 0.001). Significant differences across CTI quartiles were observed for gender, race/ethnicity, PIR, educational attainment, smoking, alcohol consumption, hypertension, diabetes, hyperlipidemia, CVD, CKD, medication use, and glycemic status (all *p* < 0.001), whereas marital status did not differ significantly (*p* = 0.073). Higher CTI quartiles were also characterized by higher BMI, SBP, DBP, FPG, HbA1c, TG, TC, UACR, serum uric acid, BUN, TyG, and CRP, together with lower HDL‐C and eGFR (all *p* < 0.001).

**Table 1:** Survey‐weighted baseline characteristics of the study population according to CTI quartiles.

Characteristics	CTI					*p*
	Total	Q1	Q2	Q3	Q4	
Age, years (SD)	46.68 ± 16.62	39.86 ± 15.06	46.56 ± 16.73	49.43 ± 16.75	50.87 ± 15.70	< 0.001
Gender, *n* (weighted %)						< 0.001
Male	5360 (49.61%)	1100 (44.99%)	1407 (53.71%)	1447 (51.81%)	1406 (47.95%)	
Female	5168 (50.39%)	1204 (55.01%)	1163 (46.29%)	1314 (48.19%)	1487 (52.05%)	
Race, *n* (weighted %)						< 0.001
Non‐Hispanic White	5507 (72.99%)	1184 (71.48%)	1375 (73.14%)	1442 (72.95%)	1506 (74.40%)	
Non‐Hispanic Black	2088 (7.31%)	329 (5.77%)	452 (6.84%)	584 (8.03%)	723 (8.61%)	
Other Race	1040 (9.46%)	251 (10.12%)	251 (9.32%)	274 (9.62%)	264 (8.77%)	
Mexican American	1893 (10.24%)	540 (12.63%)	492 (10.70%)	461 (9.40%)	400 (8.22%)	
Marital status, *n* (weighted %)						0.073
Married/living with partner	6600 (66.00%)	1395 (63.64%)	1624 (66.71%)	1772 (67.69%)	1809 (65.96%)	
Widowed/divorced/never married	3928 (34.00%)	909 (36.36%)	946 (33.29%)	989 (32.31%)	1084 (34.04%)	
PIR, *n* (weighted %)						< 0.001
≤ 1	1879 (12.39%)	366 (11.36%)	415 (10.74%)	475 (12.06%)	623 (15.39%)	
> 1, ≤ 3	4472 (36.38%)	867 (32.22%)	1074 (35.82%)	1215 (37.53%)	1316 (39.97%)	
> 3	4177 (51.23%)	1071 (56.41%)	1081 (53.44%)	1071 (50.41%)	954 (44.64%)	
Education level, *n* (weighted %)						< 0.001
Some college or above	5013 (56.31%)	1362 (66.24%)	1273 (57.44%)	1222 (53.06%)	1156 (48.49%)	
High School	5505 (43.69%)	941 (33.76%)	1296 (42.56%)	1535 (46.94%)	1733 (51.51%)	
Alcohol consumption status, *n* (weighted %)						< 0.001
Current	6976 (71.91%)	1712 (78.61%)	1785 (73.84%)	1764 (70.64%)	1715 (64.56%)	
Former	2138 (16.86%)	311 (10.78%)	475 (16.17%)	619 (18.41%)	733 (22.06%)	
Never	1414 (11.23%)	281 (10.61%)	310 (10.00%)	378 (10.95%)	445 (13.38%)	
Smoking, *n* (weighted %)						< 0.001
Former	2854 (25.82%)	467 (20.22%)	699 (25.09%)	801 (28.23%)	887 (29.74%)	
Never	5413 (50.93%)	1388 (59.13%)	1331 (51.40%)	1386 (49.56%)	1308 (43.61%)	
Current	2261 (23.25%)	449 (20.65%)	540 (23.51%)	574 (22.20%)	698 (26.65%)	
Hypertension, *n* (weighted %)						< 0.001
No	6008 (63.49%)	1751 (81.67%)	1592 (68.19%)	1420 (56.28%)	1245 (47.80%)	
Yes	4517 (36.51%)	553 (18.33%)	977 (31.81%)	1339 (43.72%)	1648 (52.20%)	
Diabetes, *n* (weighted %)						< 0.001
No	8743 (87.44%)	2205 (97.07%)	2303 (92.52%)	2345 (88.03%)	1890 (72.14%)	
Yes	1785 (12.56%)	99 (2.93%)	267 (7.48%)	416 (11.97%)	1003 (27.86%)	
Hyperlipidemia, *n* (weighted %)						< 0.001
No	2573 (26.20%)	1185 (52.23%)	791 (32.27%)	442 (15.38%)	155 (4.91%)	
Yes	7955 (73.80%)	1119 (47.77%)	1779 (67.73%)	2319 (84.62%)	2738 (95.09%)	
CVD, *n* (weighted %)						< 0.001
No	9331 (91.45%)	2185 (96.49%)	2289 (92.45%)	2432 (90.43%)	2425 (86.41%)	
Yes	1197 (8.55%)	119 (3.51%)	281 (7.55%)	329 (9.57%)	468 (13.59%)	
CKD, *n* (weighted %)						< 0.001
No	8497 (86.41%)	2075 (93.40%)	2143 (88.66%)	2196 (85.61%)	2083 (77.96%)	
Yes	1948 (13.59%)	212 (6.60%)	409 (11.34%)	543 (14.39%)	784 (22.04%)	
KDIGO CKD risk category, *n* (weighted %)						< 0.001
low risk	8497 (86.60%)	2075 (93.48%)	2143 (88.84%)	2196 (85.73%)	2083 (78.30%)	
moderate risk	1317 (9.84%)	168 (5.53%)	277 (8.25%)	375 (10.51%)	497 (15.09%)	
high risk	366 (2.21%)	26 (0.64%)	75 (1.74%)	103 (2.45%)	162 (4.03%)	
very high risk	229 (1.35%)	15 (0.36%)	47 (1.17%)	58 (1.32%)	109 (2.58%)	
Antidiabetics drug use, *n* (weighted %)						< 0.001
No	9611 (93.89%)	2252 (98.38%)	2440 (96.78%)	2535 (94.22%)	2384 (86.17%)	
Yes	915 (6.11%)	51 (1.62%)	130 (3.22%)	225 (5.78%)	509 (13.83%)	
Antihyperlipidemic drug use, *n* (weighted %)						< 0.001
No	8784 (85.84%)	2102 (93.19%)	2160 (86.73%)	2243 (82.78%)	2279 (80.64%)	
Yes	1742 (14.16%)	201 (6.81%)	410 (13.27%)	517 (17.22%)	614 (19.36%)	
Antihypertensive drug use, *n* (weighted %)						< 0.001
No	7258 (74.51%)	1939 (88.38%)	1882 (78.88%)	1814 (70.30%)	1623 (60.47%)	
Yes	3268 (25.49%)	364 (11.62%)	688 (21.12%)	946 (29.70%)	1270 (39.53%)	
Glycemic status, *n* (weighted %)						< 0.001
NGR	4740 (52.18%)	1589 (74.16%)	1355 (59.37%)	1136 (46.56%)	660 (28.65%)	
Pre‐DM	3987 (35.24%)	609 (22.90%)	946 (33.15%)	1206 (41.47%)	1226 (43.44%)	
DM	1785 (12.58%)	99 (2.94%)	267 (7.48%)	416 (11.98%)	1003 (27.91%)	
BMI, Kg/m^2^	28.42 ± 6.51	24.36 ± 4.17	27.23 ± 5.12	29.81 ± 6.25	32.35 ± 7.19	< 0.001
SBP, mmHg	121.90 ± 17.71	115.28 ± 15.33	120.75 ± 17.10	124.29 ± 17.69	127.36 ± 18.28	< 0.001
DBP, mmHg	70.48 ± 12.43	67.98 ± 11.43	69.92 ± 11.80	71.52 ± 12.46	72.52 ± 13.50	< 0.001
eGFR, mL/min/1.73 m^2^	94.27 ± 21.22	100.36 ± 19.18	94.63 ± 20.75	91.83 ± 21.07	90.25 ± 22.36	< 0.001
UACR, mg/g	32.02 ± 280.90	11.87 ± 69.71	21.72 ± 209.82	28.85 ± 234.24	65.85 ± 459.36	< 0.001
FPG, mmol/L	5.72 ± 1.56	5.18 ± 0.63	5.45 ± 0.79	5.66 ± 0.93	6.59 ± 2.59	< 0.001
HbA1c, %	5.51 ± 0.85	5.22 ± 0.40	5.36 ± 0.52	5.49 ± 0.58	5.97 ± 1.35	< 0.001
TG, mmol/L	1.59 ± 1.44	0.81 ± 0.27	1.18 ± 0.41	1.59 ± 0.57	2.78 ± 2.34	< 0.001
TC, mmol/L	5.13 ± 1.07	4.75 ± 0.91	5.02 ± 0.95	5.22 ± 1.00	5.53 ± 1.24	< 0.001
HDL, mmol/L	1.37 ± 0.41	1.60 ± 0.41	1.43 ± 0.38	1.30 ± 0.36	1.17 ± 0.33	< 0.001
LDL, mmol/L	3.06 ± 0.90	2.77 ± 0.79	3.05 ± 0.85	3.21 ± 0.91	3.21 ± 0.98	< 0.001
SCR, *μ*mol/L	77.93 ± 33.27	74.87 ± 17.74	78.33 ± 34.14	78.66 ± 33.68	79.84 ± 42.43	< 0.001
Serum uric acid, *μ*mol/L	327.43 ± 83.06	289.29 ± 71.62	321.85 ± 79.71	341.48 ± 77.84	357.04 ± 86.59	< 0.001
BUN, mmol/L	4.75 ± 1.91	4.49 ± 1.61	4.76 ± 1.76	4.80 ± 1.97	4.93 ± 2.23	< 0.001
TyG	8.68 ± 0.65	8.05 ± 0.35	8.47 ± 0.35	8.81 ± 0.37	9.38 ± 0.58	< 0.001
HGB, g/dL	14.55 ± 1.47	14.38 ± 1.46	14.61 ± 1.43	14.56 ± 1.46	14.64 ± 1.51	< 0.001
CRP, mg/L	4.15 ± 8.15	0.71 ± 0.73	2.07 ± 2.45	4.30 ± 5.30	9.51 ± 13.65	< 0.001
All‐cause mortality, *n* (weighted %)						< 0.001
No	8197 (84.18%)	2059 (93.49%)	2030 (85.76%)	2082 (81.55%)	2026 (75.91%)	
Yes	2331 (15.82%)	245 (6.51%)	540 (14.24%)	679 (18.45%)	867 (24.09%)	
CVD mortality, *n* (weighted %)						< 0.001
No	9909 (95.86%)	2243 (98.43%)	2432 (96.48%)	2571 (95.05%)	2663 (93.48%)	
Yes	619 (4.14%)	61 (1.57%)	138 (3.52%)	190 (4.95%)	230 (6.52%)	
Follow‐up time (months)	164.29 ± 50.47	169.49 ± 44.96	164.09 ± 50.09	163.90 ± 51.63	159.69 ± 54.29	< 0.001

*Note:* Continuous variables are presented as survey‐weighted means ± standard deviations (SDs), whereas categorical variables are presented as unweighted counts with survey‐weighted percentages. Percentages were calculated among participants with nonmissing data for the corresponding variable. *p* values for comparisons across CTI quartiles were obtained using survey‐weighted linear regression for continuous variables and Rao–Scott chi‐square tests for categorical variables.

Abbreviations: BMI, body mass index; BUN, blood urea nitrogen; CKD, chronic kidney disease; CRP, C‐reactive protein; CTI, C‐reactive protein–triglyceride–glucose index; DBP, diastolic blood pressure; eGFR, estimated glomerular filtration rate; FPG, fasting plasma glucose; HbA1c, glycated hemoglobin; HDL‐C, high‐density lipoprotein cholesterol; HGB, hemoglobin; LDL‐C, low‐density lipoprotein cholesterol; PIR, poverty‐income ratio; SBP, systolic blood pressure; SCR, serum creatinine; TC, total cholesterol; TG, triglycerides; TyG, triglyceride–glucose index; UACR, urine albumin‐to‐creatinine ratio.

### 3.2. Association Between CTI and All‐Cause Mortality and CVD Mortality

Survey‐weighted Cox PH regression models were used to assess the associations of CTI with all‐cause and CVD mortality (Table 2). Model 1 was unadjusted. Model 2 was designated as the primary DAG‐informed model and adjusted for age, gender, race/ethnicity, marital status, PIR, educational attainment, alcohol consumption, and smoking status. Model 3 served as an extended clinical adjustment model and additionally included hypertension, CKD, and HGB. When CTI was analyzed as a continuous variable, each 1‐unit increase in CTI was associated with higher all‐cause mortality in the primary DAG‐informed model (HR = 1.24, 95% CI: 1.18–1.31, *p* < 0.001). The association remained significant after further clinical adjustment in Model 3 (HR = 1.19, 95% CI: 1.13–1.26, *p* < 0.001). For CVD mortality, the corresponding csHRs were 1.35 (95% CI: 1.20–1.53, *p* < 0.001) in the primary model and 1.27 (95% CI: 1.13–1.44, *p* < 0.001) in the extended clinical model. In quartile analyses, compared with Q1, participants in Q4 had higher risks of both all‐cause mortality (HR = 1.54, 95% CI: 1.29–1.84, *p* < 0.001) and CVD mortality (csHR = 1.78, 95% CI: 1.27–2.49, *p* < 0.001) in the primary DAG‐informed model. These associations remained significant after additional adjustment for hypertension, CKD, and HGB in Model 3, with HRs of 1.41 (95% CI: 1.20–1.67, *p* < 0.001) for all‐cause mortality and 1.55 (95% CI: 1.12–2.14, *p* = 0.008) for CVD mortality. In the primary model, Q2 was not significantly associated with all‐cause mortality (HR = 1.19, 95% CI: 0.97–1.46, *p* = 0.105), whereas Q3 showed a borderline association (HR = 1.21, 95% CI: 1.00–1.46, *p* = 0.047). For CVD mortality, neither Q2 (csHR = 1.18, 95% CI: 0.83–1.70, *p* = 0.356) nor Q3 (csHR = 1.33, 95% CI: 0.92–1.91, *p* = 0.126) was statistically significant. Trends across CTI quartiles were significant for both all‐cause and CVD mortality in the primary model (both *p* for trend < 0.0001). Sensitivity analyses using alternative covariate specifications are presented in Table S5. For all‐cause mortality, the association comparing the highest with the lowest CTI quartile remained statistically significant across all evaluated specifications, with HRs ranging from 1.38 to 1.48 (all *p* < 0.001). For CVD mortality, the association remained statistically significant in the extended clinical adjustment model (csHR = 1.55, 95% CI: 1.12–2.14; *p* = 0.008), after additional adjustment for TC (csHR = 1.58, 95% CI: 1.14–2.18; *p* = 0.006), and when eGFR replaced CKD (csHR = 1.64, 95% CI: 1.19–2.27; *p* = 0.003). However, the estimates were attenuated and no longer statistically significant after additional adjustment for BMI (HR = 1.39, 95% CI: 0.97–1.99; *p* = 0.076) or diabetes and hyperlipidemia (HR = 1.39, 95% CI: 0.98–1.98; *p* = 0.066). These findings indicate that the association with all‐cause mortality was consistent across alternative covariate specifications, whereas the CVD mortality association was more sensitive to additional adjustment for metabolically related variables.

**Table 2:** Survey‐weighted Cox proportional hazards models for the associations of CTI with all‐cause and CVD mortality.

Outcomes	Deaths/total, *n*	Survey‐weighted mortality, (%)	Model 1		Model 2		Model 3	
			HR (95% CI)	*p*	HR (95% CI)	*p*	HR (95% CI)	*p*
All‐cause mortality								
CTI per 1‐unit increase			1.62 (1.54, 1.71)	< 0.001	1.24 (1.18, 1.31)	< 0.001	1.19 (1.13, 1.26)	< 0.001
CTI (quartile)								
Q1	245/2304	6.51%	Reference		Reference		Reference	
Q2	540/2570	14.24%	2.27 (1.85, 2.78)	< 0.001	1.19 (0.97, 1.46)	0.105	1.17 (0.96, 1.43)	0.122
Q3	679/2761	18.45%	2.94 (2.41, 3.59)	< 0.001	1.21 (1.00, 1.46)	0.047	1.17 (0.98, 1.40)	0.089
Q4	867/2893	24.09%	3.96 (3.30, 4.75)	< 0.001	1.54 (1.29, 1.84)	< 0.001	1.41 (1.20, 1.67)	< 0.001
**p** for trend			< 0.0001		< 0.0001		< 0.0001	
Cardiovascular mortality								
CTI per 1‐unit increase			1.70 (1.55, 1.87)	< 0.001	1.35 (1.20, 1.53)	< 0.001	1.27 (1.13, 1.44)	< 0.001
CTI (quartile)								
Q1	61/2304	1.57%	Reference		Reference		Reference	
Q2	138/2570	3.52%	2.32 (1.55, 3.46)	< 0.001	1.18 (0.83, 1.70)	0.356	1.15 (0.81, 1.63)	0.446
Q3	190/2761	4.95%	3.26 (2.19, 4.84)	< 0.001	1.33 (0.92, 1.91)	0.126	1.25 (0.88, 1.76)	0.208
Q4	230/2893	6.52%	4.43 (3.09, 6.35)	< 0.001	1.78 (1.27, 2.49)	< 0.001	1.55 (1.12, 2.14)	0.008
**p** for trend			< 0.0001		< 0.0001		0.0244	

*Note:* Model‐specific analytic sample sizes may differ slightly because complete‐case analysis was used for participants with missing covariate data. Model 1: unadjusted. Model 2 (primary DAG‐informed model): adjusted for age, gender, race/ethnicity, educational attainment, marital status, poverty‐income ratio, smoking status, and alcohol consumption. Model 3 (extended clinical adjustment model): Model 2 plus hypertension, chronic kidney disease, and hemoglobin. Covariates in the primary model were selected according to the minimally sufficient adjustment set identified by the directed acyclic graph. For cardiovascular mortality, Cox regression estimates are reported as cause‐specific hazard ratios (csHRs), with noncardiovascular death treated as a censoring event.

Abbreviations: CI, confidence interval; csHR, cause‐specific hazard ratio; CTI, C‐reactive protein–triglyceride–glucose index; HR, hazard ratio; Ref, reference.

### 3.3. Selection‐Bias Sensitivity Analysis

Among 35,379 adults aged ≥ 18 years, CTI was calculable for 15,261 participants (43.14%), whereas 20,118 (56.86%) lacked at least one component required for CTI calculation. Among participants without calculable CTI, 16,103 (80.04%) had CRP measurements available but lacked both fasting triglyceride and fasting glucose measurements, indicating that CTI unavailability was predominantly related to the availability of fasting laboratory measurements. Baseline comparisons showed limited measured imbalance between participants with and without available CTI, with all standardized mean differences < 0.10 (Table S1). To further evaluate selection associated with covariate completeness, an inverse‐probability‐of‐selection weighted sensitivity analysis was performed. The final IPW mortality analyses included 10,415 participants. Stabilized selection weights ranged from 0.80 to 4.15, and the Kish effective sample size based on the stabilized selection weights was approximately 9812 in the final analysis sample. Compared with Q1, the HR for all‐cause mortality in Q4 was 1.40 (95% CI, 1.17–1.66; *p* < 0.001), and the cause‐specific HR for CVD mortality was 1.52 (95% CI, 1.10–2.11; *p* = 0.012) (Table S2). These findings were materially consistent with the primary survey‐weighted analyses.

### 3.4. Assessment of the PH Assumption

In the primary all‐cause mortality diagnostic model, CTI quartiles showed no evidence of nonproportionality (*p* = 0.572), whereas sex showed evidence of a time‐varying effect (*p* = 0.007), and the global Schoenfeld test was significant (*p* = 0.0045). A conventional sensitivity model accounting for detected nonproportionality through stratification and time‐varying terms yielded a Q4‐versus‐Q1 estimate that was nearly unchanged from the corresponding conventional Cox estimate (HR 1.36 vs. 1.37). For CVD mortality, neither CTI quartiles nor the global model showed evidence of nonproportionality (*p* = 0.907 and *p* = 0.633, respectively). Similar findings were obtained for CTI in the extended clinical models. Thus, the diagnostics did not suggest CTI‐specific nonproportionality; however, the significant global test for the all‐cause model indicates that some covariate effects may vary over follow‐up, and the PH‐corrected analyses should therefore be interpreted as supportive sensitivity analyses rather than definitive validation of the survey‐weighted PH specification.

### 3.5. Competing‐Risk Sensitivity Analysis

Survey‐weighted Fine–Gray competing‐risk analyses yielded results consistent with the cause‐specific Cox analyses. In the primary DAG‐informed model, the sHR was 1.35 (95% CI, 1.20–1.53; *p* < 0.001) per 1‐unit increase in CTI and 1.82 (95% CI, 1.28–2.60; *p* < 0.001) for Q4 versus Q1. In the extended clinical adjustment model, the corresponding sHRs were 1.28 (95% CI, 1.14–1.45; *p* < 0.001) and 1.62 (95% CI, 1.15–2.28; *p* = 0.006), respectively (Table S7).

### 3.6. RCS and Threshold Effect Analysis

The RCS analysis shown in Figure 2 suggested nonlinear associations between CTI and both mortality outcomes after multivariable adjustment. The subsequent two‐piecewise analyses were exploratory and used conventional unweighted Cox regression for data‐driven likelihood‐based breakpoint estimation and model comparison; therefore, the linear‐model estimates in Table 3 differ slightly from the corresponding survey‐weighted estimates reported in Table 2. The estimated breakpoints are reported as point estimates because breakpoint confidence intervals were not derived in the original analysis. For all‐cause mortality, the estimated inflection point was 8.165. Below this point, CTI was not significantly associated with mortality (HR = 0.987, 95% CI: 0.881–1.106, *p* = 0.825), whereas above the inflection point the association was significant (HR = 1.273, 95% CI: 1.181–1.373, *p* < 0.001). The conventional likelihood‐ratio comparison yielded *p* = 0.002; because the breakpoint was selected from the data, this *p* value was interpreted descriptively and the finding was regarded as exploratory evidence of a possible change in slope rather than confirmation of a fixed biological threshold. For CVD mortality, the estimated inflection point was 8.070; the HRs below and above this point were 0.999 (95% CI: 0.784–1.272, *p* = 0.990) and 1.354 (95% CI: 1.185–1.547, *p* < 0.001), respectively. The likelihood‐ratio comparison was not statistically significant (*p* = 0.067), providing insufficient evidence that the two‐piecewise model fit CVD mortality better than the linear model.

**Figure 2:**
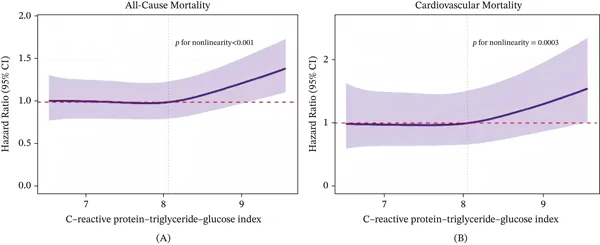
Restricted cubic spline analyses of the associations of CTI with (A) all‐cause mortality and (B) CVD mortality. Note: The solid line represents the estimated hazard ratio, and the shaded area represents the corresponding 95% confidence interval. The horizontal dashed line indicates HR = 1. The vertical dashed lines indicate the exploratory change‐point estimates from the two‐piecewise Cox analyses (8.165 for all‐cause mortality and 8.070 for CVD mortality). Models were adjusted for age, gender, race/ethnicity, marital status, poverty‐income ratio, educational attainment, alcohol consumption, smoking status, hypertension, chronic kidney disease, and hemoglobin. CI, confidence interval; CTI, C‐reactive protein–triglyceride–glucose index; HR, hazard ratio.

**Table 3:** Exploratory two‐piecewise Cox regression analyses of the associations of CTI with all‐cause and cardiovascular mortality.

Outcomes	All‐cause mortality		CVD mortality	
	HR (95% CI)	*p*	csHR (95% CI)	*p*
CTI				
Fitting by standard linear model	1.163 (1.105–1.224)	< 0.0001	1.237 (1.122–1.363)	< 0.0001
Fitting by two‐piecewise linear model				
Inflection point (K)	8.165		8.070	
CTI < K	0.987 (0.881–1.106)	0.825	0.999 (0.784–1.272)	0.990
CTI > K	1.273 (1.181–1.373)	< 0.001	1.354 (1.185–1.547)	< 0.001
*p* for log‐likelihood ratio test	0.002		0.067	

*Note:* Values are hazard ratios (HRs) with 95% confidence intervals (CIs). The threshold analyses were performed using conventional Cox proportional hazards regression. The standard and two‐piecewise Cox proportional hazards models were fitted in the same complete‐case analytic sample with identical covariate adjustment for age, gender, race/ethnicity, educational attainment, marital status, poverty‐income ratio, smoking status, alcohol consumption, hypertension, chronic kidney disease, and hemoglobin. Conventional Cox regression was used because the likelihood‐based breakpoint search and likelihood‐ratio comparison employed for the threshold analysis are not directly available for survey‐weighted Cox regression. Therefore, the estimates from the standard Cox proportional hazards model shown in this table differ slightly from the survey‐weighted continuous‐CTI estimates reported in Table 2. Because K was selected using a data‐driven procedure, the likelihood‐ratio *p* values are descriptive rather than confirmatory. Confidence intervals for K were not estimated in the original analysis.

### 3.7. Kaplan–Meier Survival and Time‐Dependent Discrimination Analyses

Kaplan–Meier curves by CTI quartile are shown in Figure 3. All‐cause survival differed significantly across CTI quartiles (log‐rank *p* < 0.001), with progressively lower survival observed at higher CTI levels. For CVD mortality, Kaplan–Meier analysis treating non‐CVD deaths as censored observations likewise demonstrated significant differences in cause‐specific survival across CTI quartiles (log‐rank *p* < 0.001). These curves should be interpreted as cause‐specific survival curves rather than estimates of the cumulative incidence of CVD death. Time‐dependent discrimination of CTI, CRP, and TyG was evaluated at prespecified 5‐, 10‐, and 15‐year time points (Table 4 and Figure 4). For all‐cause mortality, the AUCs for CTI were 0.609 (95% CI: 0.588–0.630), 0.602 (95% CI: 0.587–0.617), and 0.601 (95% CI: 0.586–0.616) at 5, 10, and 15 years, respectively. CTI did not differ significantly from CRP at 5 or 10 years (*p* = 0.947 and *p* = 0.266, respectively), but showed a higher AUC than CRP at 15 years (*p* < 0.001). CTI showed higher AUCs than TyG at all three time points (*p* < 0.001, *p* < 0.001, and *p* = 0.008, respectively). For CVD mortality, the corresponding AUCs for CTI were 0.618 (95% CI: 0.578–0.657), 0.618 (95% CI: 0.591–0.645), and 0.599 (95% CI: 0.574–0.624). The AUCs of CTI and CRP did not differ significantly at 5, 10, or 15 years (*p* = 0.533, *p* = 0.630, and *p* = 0.171, respectively), whereas CTI showed higher AUCs than TyG at all three time points (*p* < 0.001, *p* < 0.001, and *p* = 0.033, respectively). Overall, the time‐dependent discriminatory performance of CTI was modest.

**Figure 3:**
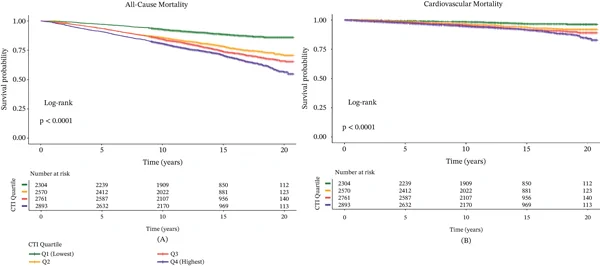
Kaplan–Meier curves for (A) all‐cause survival and (B) cause‐specific survival free of cardiovascular death according to CTI quartiles. Note: The curves display the full available follow‐up period, with numbers at risk shown at 0, 5, 10, 15, and 20 years. In Panel B, deaths from non‐CVD causes were treated as censored observations; therefore, the curves represent cause‐specific survival rather than the cumulative incidence of CVD death. Overall differences among CTI quartiles were assessed using the log‐rank test.

**Table 4:** Time‐dependent discriminative performance of CTI, CRP, and TyG for all‐cause and cardiovascular mortality.

Marker	5‐year AUC (95% CI)	*p* vs. CTI ^∗^	10‐year AUC (95% CI)	*p* vs. CTI ^∗^	15‐year AUC (95% CI)	*p* vs. CTI ^∗^
All‐cause mortality						
CTI	0.609 (0.588–0.630)	Reference	0.602 (0.587–0.617)	Reference	0.601 (0.586–0.616)	Reference
CRP	0.613 (0.591–0.635)	0.947	0.593 (0.577–0.608)	0.266	0.578 (0.563–0.593)	< 0.001
TyG	0.558 (0.535–0.581)	< 0.001	0.568 (0.553–0.584)	< 0.001	0.587 (0.571–0.602)	0.008
Cardiovascular mortality						
CTI	0.618 (0.578–0.657)	Reference	0.618 (0.591–0.645)	Reference	0.599 (0.574–0.624)	Reference
CRP	0.599 (0.560–0.639)	0.533	0.607 (0.580–0.635)	0.630	0.581 (0.556–0.607)	0.171
TyG	0.569 (0.527–0.611)	< 0.001	0.574 (0.545–0.603)	< 0.001	0.578 (0.553–0.604)	0.033

*Note:* Time‐dependent cumulative/dynamic AUCs were estimated at prespecified 5‐, 10‐, and 15‐year time points using inverse probability of censoring weighting (IPCW) as implemented in the timeROC package. Pointwise 95% confidence intervals were derived from the iid representation of the AUC estimator using its asymptotic normal distribution. For cardiovascular mortality, noncardiovascular death was treated as a competing event.

Abbreviations: AUC, area under the curve; CI, confidence interval; CRP, C‐reactive protein; CTI, C‐reactive protein–triglyceride–glucose index; CVD, cardiovascular disease; TyG, triglyceride–glucose index.

^∗^ *p* values compare the time‐dependent AUC of CRP or TyG with that of CTI at the corresponding time point using the iid‐representation‐based comparison test implemented in timeROC::compare. These standalone timeROC analyses were conducted using conventional unweighted methods. The IPCW weights accounted for censoring and were distinct from the NHANES survey sampling weights.

**Figure 4:**
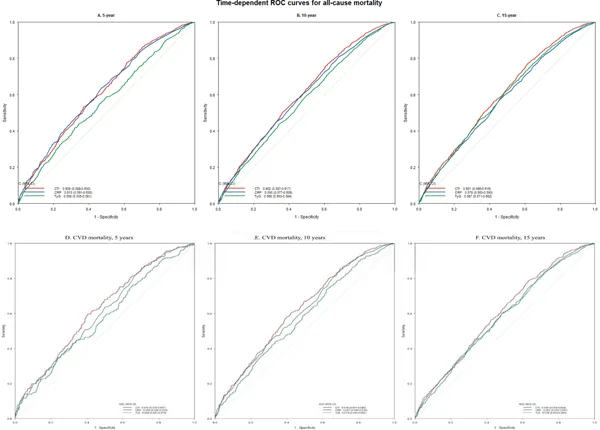
Time‐dependent receiver operating characteristic curves for CTI, CRP, and TyG for all‐cause and cardiovascular mortality. Note: Panels (A–C) show discrimination for all‐cause mortality at 5, 10, and 15 years, respectively, whereas Panels (D–F) show discrimination for cardiovascular mortality at the corresponding time points. For cardiovascular mortality, noncardiovascular death was treated as a competing event in the time‐dependent AUC analysis. AUC, area under the curve; CRP, C‐reactive protein; CTI, C‐reactive protein–triglyceride–glucose index; CVD, cardiovascular disease; TyG, triglyceride–glucose index.

### 3.8. Incremental Predictive Performance of CTI

Because model fitting and performance evaluation were conducted in the same sample, these estimates represent apparent performance. Incremental prediction analyses were conducted in 10,279 participants with complete data for the prediction‐oriented base model. Addition of CTI resulted in only small changes in predictive performance beyond established clinical risk factors (Table 5). For all‐cause mortality, Harrell’s C‐index increased from 0.8604 for the base model to 0.8619 after addition of CTI (*Δ*C − index = 0.0014, 95% CI: 0.0007–0.0024). The corresponding 5‐, 10‐, and 15‐year Brier scores changed from 0.046440, 0.080030, and 0.093030 to 0.046199, 0.079719, and 0.092920, respectively, representing only small reductions in prediction error. For CVD mortality, the 5‐year time‐dependent AUC increased from 0.8714 to 0.8741 (*Δ*AUC = 0.0028, 95% CI: −0.0004–0.0059), the 10‐year AUC increased from 0.8594 to 0.8620 (*Δ*AUC = 0.0026, 95% CI: 0.0005–0.0048), and the 15‐year AUC changed from 0.8477 to 0.8479 (*Δ*AUC = 0.0002, 95% CI: −0.0018–0.0022). The corresponding Brier scores were 0.015078 versus 0.015058 at 5 years, 0.030446 versus 0.030380 at 10 years, and 0.049000 versus 0.049047 at 15 years for the base and CTI‐augmented models, respectively. Thus, changes in overall prediction error were negligible. Calibration was broadly similar between the base and CTI‐augmented models at 5, 10, and 15 years for both outcomes (Figure S6). Decision curve analyses likewise showed largely overlapping net‐benefit curves across the evaluated threshold probabilities, with no substantial or consistent gain in clinical net benefit after addition of CTI (Figure S7). Overall, CTI provided only limited incremental predictive improvement beyond established clinical risk factors.

**Table 5:** Incremental predictive performance after adding CTI to a prediction‐oriented base model.

Outcome	Conventional model	*C* *o* *n* *v* *e* *n* *t* *i* *o* *n* *a* *l* *m* *o* *d* *e* *l* + *C* *T* *I*	Difference (95% CI)
All‐cause mortality			
Harrell C‐index	0.8604	0.8619	+0.0014 (0.0007 to 0.0024)
5‐year Brier score	0.046440	0.046199	−0.000241 (−0.000473 to −0.000010)
10‐year Brier score	0.080030	0.079719	−0.000311 (−0.000731 to 0.000110)
15‐year Brier score	0.093030	0.092920	−0.000109 (−0.000712 to 0.000493)
CVD mortality			
5‐year time‐dependent AUC	0.8714	0.8741	+0.0028 (−0.0004 to 0.0059)
10‐year time‐dependent AUC	0.8594	0.8620	+0.0026 (0.0005 to 0.0048)
15‐year time‐dependent AUC	0.8477	0.8479	+0.0002 (−0.0018 to 0.0022)
5‐year Brier score	0.015078	0.015058	−0.000020 (−0.000074 to 0.000034)
10‐year Brier score	0.030446	0.030380	−0.000065 (−0.000170 to 0.000040)
15‐year Brier score	0.049000	0.049047	+0.000046 (−0.000108 to 0.000200)

*Note:* The prediction‐oriented conventional model included age, gender, race/ethnicity, smoking status, hypertension, diabetes mellitus, body mass index, total cholesterol, HDL cholesterol, and chronic kidney disease. The extended prediction model additionally included continuous CTI. Both models were fitted in the same complete‐case prediction sample. For all‐cause mortality, discrimination was summarized using Harrell′s C‐index. For CVD mortality, discrimination was evaluated using time‐dependent AUCs at 5, 10, and 15 years in a competing‐risk framework, with non‐CVD death treated as a competing event. Differences were calculated as the value for the conventional model plus CTI minus that for the conventional model using unrounded estimates. The 95% CI for the difference in Harrell′s C‐index was estimated using 2000 paired bootstrap resamples. Confidence intervals for differences in time‐dependent AUCs and Brier scores were obtained using influence‐function‐based inference. Lower Brier scores indicate better overall prediction accuracy. Because model development and performance assessment were performed in the same complete‐case sample, all reported performance measures represent apparent performance; no cross‐validation or optimism‐corrected internal validation was performed.

Abbreviations: CTI, C‐reactive protein–triglyceride–glucose index; CVD, cardiovascular disease.

### 3.9. Subgroup Analyses

In exploratory subgroup analyses, the association between continuous CTI and all‐cause mortality differed by age (*p* for interaction < 0.001), with a stronger association among participants aged ≤ 60 years (HR 1.44, 95% CI 1.29–1.61) than among those aged > 60 years (HR 1.00, 95% CI 0.93–1.08). No clear evidence of interaction was observed for the other evaluated subgroups. For CVD mortality, interaction with age was also observed (*p* for interaction < 0.001), with a stronger association among participants aged ≤ 60 years (csHR 1.48, 95% CI 1.18–1.86) than among those aged > 60 years (csHR 1.07, 95% CI 0.93–1.24). A nominal interaction by race/ethnicity was observed (*p* for interaction = 0.030); however, this finding should be interpreted cautiously because multiple interaction tests were performed without multiplicity adjustment and some racial/ethnic strata contained relatively few CVD deaths. The subgroup estimates and interaction tests are presented in Figure 5.

**Figure 5:**
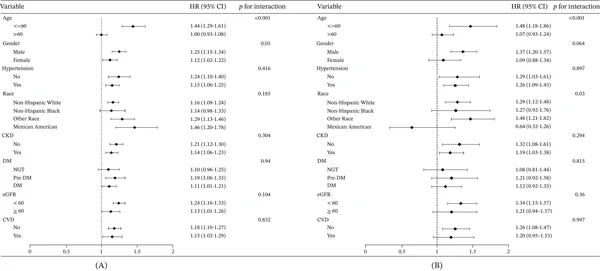
Subgroup analyses of the association between continuous CTI and mortality. Note: Panel (A) shows all‐cause mortality and Panel (B) shows CVD mortality. HRs and 95% CIs are reported per 1‐unit increase in CTI. Models were adjusted according to the extended clinical model, excluding the corresponding stratifying variable; CKD was additionally omitted from eGFR‐stratified analyses. For CVD mortality, non‐CVD deaths were censored and estimates are reported as cause‐specific HRs. *p* values for interaction were obtained from multiplicative interaction terms. Because no multiplicity adjustment was applied, subgroup analyses were considered exploratory. Abbreviations: CI, confidence interval; CKD, chronic kidney disease; CTI, C‐reactive protein–triglyceride–glucose index; CVD, cardiovascular disease; eGFR, estimated glomerular filtration rate; HR, hazard ratio.

## 4. Discussion

In this nationally representative sample of U.S. adults with mortality follow‐up, higher CTI was associated with greater hazards of all‐cause and CVD mortality after adjustment for prespecified covariates. These associations were generally robust across additional adjustment and sensitivity analyses, although the association with CVD mortality showed greater sensitivity to adjustment for metabolically related factors. RCS and exploratory two‐piecewise analyses suggested a possible change in slope for all‐cause mortality, whereas evidence for a distinct change point in CVD mortality was inconclusive. Because breakpoint selection was data driven and interval estimates for the breakpoints were not available, these findings should not be interpreted as validation of a fixed biological threshold. Although CTI showed statistically significant prognostic associations with mortality, its standalone and incremental discriminative performance was modest. Importantly, the incremental prediction analyses showed that adding CTI to a prediction‐oriented model containing established clinical risk factors produced only small improvements in discrimination. Changes in Brier scores were minimal, calibration remained broadly similar between models, and decision curve analyses showed little consistent gain in clinical net benefit. Thus, although the association between CTI and mortality persisted after multivariable adjustment, its incremental contribution to prediction was quantitatively small. The small changes in model performance suggest that CTI may complement, rather than materially improve, conventional clinical risk assessment.

Originally proposed by Ruan et al. [34] CTI serves as an integrated biomarker derived from C‐reactive protein and the TyG index, thereby enabling a combined assessment of systemic inflammatory status and IR [46], two closely interconnected pathways involved in adverse clinical outcomes. Early studies demonstrated its prognostic value in cancer populations [35], and subsequent investigations extended its relevance to cancer mortality, stroke risk, and incident CVD [5, 44, 47, 48]. More recently, however, several population‐based analyses have directly examined CTI in relation to mortality among U.S. adults. Sun et al. analyzed NHANES 2001–2010 and 2015–2018 and reported positive associations of higher CTI with all‐cause mortality, cardiovascular mortality, and prevalent CVD [49]. Yu et al., using NHANES 2001–2010, similarly found higher all‐cause and cardiovascular mortality with increasing CTI and reported nonlinear associations in restricted cubic spline analyses [50]. In addition, Li et al. examined NHANES 1999–2010 and observed positive associations of CTI with both mortality outcomes, with stronger evidence for nonlinearity in all‐cause mortality than in cardiovascular mortality [51]. Thus, the present study should be viewed as extending an emerging body of population‐based evidence rather than as the first demonstration of an association between CTI and mortality in U.S. adults. Its contribution lies principally in the comprehensive analytic framework used to examine the robustness, shape, and potential clinical relevance of these associations, including fasting‐subsample survey weighting, DAG‐informed confounding control, inverse‐probability‐of‐selection weighting, formal proportional‐hazards diagnostics, competing‐risk analyses for CVD mortality, formal threshold evaluation, time‐dependent discrimination analyses, and assessment of incremental predictive performance.

The associations were attenuated after adjustment for the DAG‐informed set of demographic and lifestyle confounders and were further attenuated after additional adjustment for selected clinical factors. Sensitivity analyses using alternative covariate specifications showed that the association with all‐cause mortality remained statistically significant across all evaluated models. For CVD mortality, the direction of association was generally preserved, although the estimates were attenuated and the confidence intervals crossed the null after additional adjustment for BMI or diabetes and hyperlipidemia. Alternative adjustment strategies produced broadly stable estimates for all‐cause mortality, whereas estimates for CVD mortality were more attenuated and less precise after inclusion of additional metabolic variables. RCS analyses suggested nonlinear associations between CTI and both mortality outcomes. In the subsequent two‐piecewise analysis, the estimated inflection points were 8.165 for all‐cause mortality and 8.070 for CVD mortality. For all‐cause mortality, CTI was not significantly associated with risk below the inflection point, whereas risk increased significantly above it, and the likelihood‐ratio test supported the two‐piecewise specification (*p* = 0.002). For CVD mortality, CTI was likewise not significantly associated with risk below the estimated inflection point and was positively associated above it; however, the likelihood‐ratio test was not significant *(* *p* = 0.067). Therefore, the threshold finding for CVD mortality should be interpreted cautiously, because the two‐piecewise model did not demonstrate a statistically significant improvement over the linear model. Because CTI combines CRP, TG, and FPG, higher CTI levels may indicate a state characterized by systemic inflammation, impaired insulin sensitivity, and adverse lipid metabolism. Existing evidence suggests that inflammation and IR may jointly promote atherosclerosis through multiple mechanisms, including endothelial dysfunction, oxidative stress, inflammatory cell adhesion and infiltration, plaque destabilization, and progressive fibrosis, thereby contributing to cardiovascular complications and mortality [52–55]. Further analysis in conjunction with baseline characteristics reveals that, as the CTI increases, participants′ BMI, SBP, DBP, FPG, HbA1c, TG, TC, UACR, UA and CRP levels increased, whereas HDL‐C and eGFR decreased; simultaneously, the prevalence of diabetes, dyslipidemia, CKD and a history of CVD increases. This suggests that an elevated CTI is not an isolated laboratory phenomenon, but rather a concentrated manifestation of a multisystem adverse cardiometabolic phenotype within a single composite indicator.

Kaplan–Meier survival curves showed distinct survival patterns across CTI quartiles, with significant between‐group differences for both all‐cause and CVD mortality (log‐rank *p* < 0.001). In time‐dependent ROC analyses, CTI showed modest discrimination over 5‐, 10‐, and 15‐year horizons. For all‐cause mortality, CTI was comparable with CRP at 5 and 10 years but had a higher AUC at 15 years; CTI also showed higher AUCs than TyG at all three time points. For CVD mortality, CTI and CRP had comparable AUCs at all prespecified time points, whereas CTI showed higher AUCs than TyG. These results indicate that CTI should not be viewed as a strong standalone predictive marker; rather, its potential value may lie in integrating inflammatory and metabolic information for adjunctive risk characterization. CRP, TG, and FPG reflect systemic inflammation, dyslipidemia, and metabolic disturbance, respectively, all of which are closely related to metabolic syndrome and cardiometabolic disorders. IR is a central pathophysiological feature underlying many cardiometabolic disorders [56–58]. This contributes to metabolic dysregulation; IR fuels the advance of atherosclerosis via multiple pathways, such as elevated cholesterol levels, imbalanced lipid profiles, high blood pressure, and issues with the inner lining of blood vessels and smooth muscle function [14], elevated levels of oxidative stress [59], dysregulated lipid metabolism and the buildup of lipotoxic agents [60], and the prolonged activation of several inflammatory pathways [33]; at the same time, systemic inflammation can also exacerbate endothelial damage, reduce the bioavailability of nitric oxide, and promote thrombosis and plaque instability [61–64], ultimately accelerating the progression of atherosclerosis and causing multiorgan damage, thereby significantly increasing the risk of cardiovascular and cerebrovascular events. Previous mechanistic investigations indicate that low‐grade inflammation and IR may collaborate to initiate inflammatory processes in endothelial cells, including activating the NF‐*κ*B pathway. Through its effects on adhesion molecule expression, particularly VCAM‐1 and ICAM‐1, these processes may increase monocyte adhesion and foam cell development, which in turn helps contribute to the initiation and progression of atherosclerosis [52, 53]. In the context of ongoing chronic inflammation, macrophage polarization and cytokine release, especially IL‐6 and TNF‐*α*, may further participate in the MMP‐9‐ and MMP‐2‐mediated degradation of the fibrous cap, which may increase neovascularization within the plaque [54]. Furthermore, the AGEs‐RAGE signaling pathway associated with impaired glucose metabolism may also contribute to the progression of vascular damage through mitochondrial dysfunction, endothelial cell apoptosis and fibrotic processes [55]. The observed nonlinear pattern is biologically plausible in the context of combined inflammatory and metabolic dysregulation, but CTI was measured only once at baseline. Accordingly, the present study cannot establish persistent exposure, a discrete biological threshold, reversibility, or treatment effects. The observed curve shape should be regarded as a descriptive epidemiologic pattern and not as evidence of a discrete biological transition.

Subgroup analyses indicated that CTI remained positively associated with all‐cause mortality in most stratified populations. Notably, age significantly modified this association, with a relatively stronger effect observed among participants aged ≤ 60 years. No significant interactions were observed for gender, race/ethnicity, hypertension, CKD, glycemic status, baseline CVD, or eGFR. Exploratory subgroup analyses suggested effect modification by age for both all‐cause and CVD mortality. A nominal interaction by race/ethnicity was also observed for CVD mortality; however, this finding should be interpreted cautiously because of multiple interaction testing and the limited number of CVD mortality in some racial/ethnic strata. These findings support an association between higher CTI and poorer survival, although the magnitude of this association may differ by age. Nevertheless, the subgroup findings warrant cautious interpretation, as residual confounding, interindividual heterogeneity, and the utilization of a singular baseline measurement of CTI may have nevertheless impacted the observed associations.

## 5. Limitations

Several limitations should be considered when interpreting these observational findings. Residual confounding remains possible, and the study design does not permit causal conclusions. Second, CTI was assessed at a single baseline examination and may not capture changes in inflammatory or metabolic status over time. Third, CTI availability depended substantially on the NHANES fasting‐subsample design. Although the appropriate fasting‐subsample weights and an inverse‐probability‐of‐selection sensitivity analysis were used, residual selection related to unmeasured characteristics cannot be excluded. Fourth, the exploratory change‐point analyses used data‐driven breakpoint selection in conventional unweighted Cox models. Breakpoint confidence intervals were not estimated in the original analysis. Accordingly, conventional likelihood‐ratio *p* values from the data‐driven change‐point analysis were treated as descriptive rather than confirmatory. Fifth, the standalone time‐dependent ROC analyses and incremental prediction analyses were conducted using conventional unweighted methods and should not be interpreted as survey‐weighted national prediction performance. The prediction metrics were evaluated in the same complete‐case sample used for model fitting and therefore represent apparent performance; the paired bootstrap analysis quantified uncertainty in the change in Harrell′s C‐index but did not provide optimism‐corrected internal validation. Sixth, subgroup and interaction analyses involved multiple comparisons without multiplicity adjustment and should be regarded as exploratory. External validation and formal internal validation of prediction performance are warranted before clinical application.

## 6. Conclusion

In U.S. adults, higher CTI was associated with all‐cause and CVD mortality, although its added predictive value was limited. The association persisted after adjustment for demographic, lifestyle, and selected clinical covariates. Exploratory analyses were compatible with a change in slope for all‐cause mortality, whereas the evidence for a distinct threshold in CVD mortality was less definitive. CTI showed modest time‐dependent discrimination and may be useful as an adjunctive composite marker reflecting combined inflammatory and metabolic burden. Further studies should externally validate the limited incremental predictive value observed in the present analysis and determine whether CTI provides clinically useful risk information in specific populations.

## Author Contributions

Xin Wang, Siyuan Ma, and Xinrui Hai: conceptualization, data curation, formal analysis, investigation, methodology, software, validation, visualization, and writing—original draft. Ali Ma, Peng Wu, and Xiaolan Liang: investigation and writing—original draft. Yu Hao: writing—review and editing, supervision. Ning Yan and Libo Yang: investigation, supervision, validation, writing—review and editing. Xin Wang, Siyuan Ma, and Xinrui Hai contributed equally to this work and share first authorship. Ning Yan and Libo Yang are co‐corresponding authors.

## Funding

This study was supported by the Natural Science Foundation of Ningxia Province (10.13039/501100004772; 2026AAC031043); Ningxia Health Commission Research Project (2025‐NWZC‐A003); Ningxia Medical University Intramural Research Project (XM2019049); and the Key Research and Development Program Project of Ningxia (2026BEG02063).

## Disclosure

All authors have read and approved the final version of the manuscript.

## Conflicts of Interest

The authors declare no conflicts of interest.

## Supporting information


**Supporting Information** Additional supporting information can be found online in the Supporting Information section. The supplementary material is provided in a separate file and includes the following tables and figures. Table S1: Baseline characteristics of participants with and without sufficient laboratory data to calculate the C‐reactive protein–triglyceride–glucose index (CTI), including standardized mean differences between the groups. Table S2: Inverse‐probability‐of‐selection weighted sensitivity analysis comparing the associations of CTI with all‐cause and cardiovascular mortality against the primary survey‐weighted estimates. Table S3: Generalized variance inflation factors for model covariates, used to assess multicollinearity. Table S4: Correlations between CTI and selected metabolic and renal variables, providing context for covariate selection. Table S5: Sensitivity analyses using alternative covariate specifications for the associations of CTI with all‐cause and cardiovascular mortality. Table S6: Proportional hazards diagnostics for the primary and extended Cox models, together with sensitivity analyses accounting for identified nonproportional hazards. Supplementary Table S7: Survey‐weighted Fine–Gray competing‐risk analyses of the association between CTI and cardiovascular mortality. Figure S1: Directed acyclic graph showing the hypothesized relationships among CTI, mortality, and potential confounders, and identifying the covariates selected for the primary adjustment model. Figure S2: Scaled Schoenfeld residual plots assessing the proportional hazards assumption in the primary model for all‐cause mortality. Figure S3: Scaled Schoenfeld residual plots assessing the proportional hazards assumption in the primary model for cardiovascular mortality. Figure S4: Scaled Schoenfeld residual plots assessing the proportional hazards assumption in the extended clinical adjustment model for all‐cause mortality. Figure S5: Scaled Schoenfeld residual plots assessing the proportional hazards assumption in the extended clinical adjustment model for cardiovascular mortality. Figure S6: Calibration plots comparing predicted and observed risks at 5, 10, and 15 years for the base clinical model and the model additionally including CTI, for both mortality outcomes. Figure S7: Decision curve analyses comparing the net benefit of the base clinical model and the model additionally including CTI across risk thresholds at 5, 10, and 15 years for both mortality outcomes.

## Data Availability

The data that support the findings of this study are available in National Center for Health Statistics at https://wwwn.cdc.gov/nchs/nhanes/Default.aspx. These data were derived from the following resources available in the public domain: NHANES 1999–2010, https://wwwn.cdc.gov/nchs/nhanes/continuousnhanes/default.aspx?BeginYear=1999.
