# Supplementary material for: Association of the C‐Reactive Protein–Triglyceride–Glucose Index With All‐Cause and Cardiovascular Mortality in U.S. Adults: Insights From NHANES 1999–2010
Source: Cardiovasc Ther. 2026 Oct 8;2026:4209201. doi: 10.1155/cdr/4209201 (PMC13646895; doi:10.1155/cdr/4209201)
Supplement: Supplementary file 1 [file CDR-2026-4209201-s001.docx]

**SUPPLEMENTAL MATERIAL**

**Supplementary Table S1. Baseline characteristics: CTI available vs unavailable**

| Characteristics | CTI unavailable (n=20,118) | CTI available (n=15,261) | P value | SMD |
| --- | --- | --- | --- | --- |
| Age, years | 47.63 ± 20.29 | 47.15 ± 19.73 | 0.025 | 0.024 |
| Sex, n (%) |  |  | 0.297 | 0.011 |
| Female | 10,495 (52.2) | 7,875 (51.6) |  |  |
| Male | 9,623 (47.8) | 7,386 (48.4) |  |  |
| Race/ethnicity, n (%) |  |  | 0.017 | 0.034 |
| Mexican American | 4,225 (21.0) | 3,019 (19.8) |  |  |
| Non-Hispanic White | 9,534 (47.4) | 7,261 (47.6) |  |  |
| Other Race | 2,166 (10.8) | 1,653 (10.8) |  |  |
| Non-Hispanic Black | 4,193 (20.8) | 3,328 (21.8) |  |  |
| Marital status, n (%) |  |  | <0.001 | 0.055 |
| Married/Living with Partner | 10,845 (55.8) | 8,641 (58.6) |  |  |
| Widowed/Divorced/Never married | 8,585 (44.2) | 6,115 (41.4) |  |  |
| Poverty-income ratio | 2.49 ± 1.62 | 2.53 ± 1.62 | 0.018 | 0.027 |
| BMI, kg/m² | 28.71 ± 6.56 | 28.65 ± 6.51 | 0.467 | 0.008 |
| Smoking status, n (%) |  |  | 0.033 | 0.030 |
| Never | 8,813 (52.6) | 7,278 (52.1) |  |  |
| Former | 4,210 (25.1) | 3,679 (26.4) |  |  |
| Current | 3,736 (22.3) | 3,005 (21.5) |  |  |
| Alcohol consumption, n (%) |  |  | 0.313 | 0.018 |
| Never | 2,224 (14.8) | 1,840 (14.2) |  |  |
| Former | 3,075 (20.5) | 2,705 (20.9) |  |  |
| Current | 9,722 (64.7) | 8,403 (64.9) |  |  |
| Hypertension, n (%) |  |  | 0.685 | 0.005 |
| No | 9,923 (59.2) | 8,238 (59.0) |  |  |
| Yes | 6,840 (40.8) | 5,734 (41.0) |  |  |
| Diabetes mellitus, n (%) |  |  | <0.001 | 0.088 |
| No | 13,766 (85.3) | 10,995 (82.1) |  |  |
| Yes | 2,364 (14.7) | 2,400 (17.9) |  |  |
| Chronic kidney disease, n (%) |  |  | 0.002 | 0.036 |
| No | 11,889 (79.8) | 11,211 (81.2) |  |  |
| Yes | 3,013 (20.2) | 2,594 (18.8) |  |  |
| Cardiovascular disease, n (%) |  |  | <0.001 | 0.042 |
| No | 16,162 (87.4) | 12,410 (88.8) |  |  |
| Yes | 2,320 (12.6) | 1,566 (11.2) |  |  |

Continuous variables are presented as mean ± SD and categorical variables as unweighted counts (percentages among participants with nonmissing data for the corresponding characteristic). Standardized mean differences (SMDs) were calculated to quantify between-group imbalance. An absolute SMD <0.10 was considered indicative of limited imbalance. Because of the large sample size, P values were interpreted together with SMDs.
Abbreviations: BMI, body mass index; CTI, C-reactive protein-triglyceride-glucose index; SMD, standardized mean difference.

**Supplementary Table S2. Inverse-probability-of-selection weighted sensitivity analysis**

| Outcome | CTI comparison | Standard survey-weighted model (95% CI) | IPW sensitivity analysis (95% CI) | P value |
| --- | --- | --- | --- | --- |
| All-cause mortality | Q4 vs Q1 | 1.41 (1.20–1.67) | 1.40 (1.17–1.66) | <0.001 |
| Cardiovascular mortality | Q4 vs Q1 | 1.55 (1.12–2.14) | 1.52 (1.10–2.11) | 0.012 |

Abbreviations: CI, confidence interval; CTI, C-reactive protein–triglyceride–glucose index; CVD, cardiovascular disease; HR, hazard ratio; IPW, inverse-probability weighting; csHR, cause-specific hazard ratio.
**Note:** The IPW sensitivity analysis used combined weights equal to the NHANES fasting-subsample survey weight multiplied by the stabilized inverse-probability-of-selection weight. The original NHANES strata (SDMVSTRA) and primary sampling units (SDMVPSU) were retained. The selection model included age, age squared, sex, race/ethnicity, educational attainment, and NHANES survey cycle. Mortality models were adjusted for age, sex, race/ethnicity, educational attainment, marital status, poverty-income ratio, smoking status, alcohol consumption, hypertension, chronic kidney disease, and hemoglobin. Among participants included in the final IPW mortality analyses, stabilized inverse-probability-of-selection weights ranged from 0.803 to 4.154, and the Kish effective sample size based on these weights was 9,812. The analyses included 10,415 participants, including 2,296 all-cause deaths and 615 cardiovascular deaths. For cardiovascular mortality, estimates are reported as cause-specific hazard ratios (csHRs).

**Supplementary Table S3. Generalized variance inflation factors**

| **Covariate** | **GVIF** | **df** | **GVIF^(1/(2×Df))** |
| --- | --- | --- | --- |
| Age | 1.220 | 1 | 1.105 |
| Sex | 1.085 | 1 | 1.042 |
| Race/ethnicity | 1.197 | 3 | 1.030 |
| Educational attainment | 1.261 | 1 | 1.123 |
| Marital status | 1.100 | 1 | 1.049 |
| Poverty-income ratio | 1.344 | 1 | 1.159 |
| Smoking status | 1.309 | 2 | 1.070 |
| Alcohol consumption | 1.260 | 2 | 1.060 |

**Abbreviations:** DAG, directed acyclic graph; GVIF, generalized variance inflation factor; Df, degrees of freedom. GVIFs were assessed for variables included in the primary DAG-informed model, comprising age, sex, race/ethnicity, educational attainment, marital status, poverty-income ratio, smoking status, and alcohol consumption. For variables with more than one degree of freedom, GVIF^(1/(2×Df)) is reported to facilitate comparison across covariates. Values close to 1 indicate little evidence of problematic multicollinearity.

Supplementary Table S4. Correlations between CTI and selected metabolic/renal variables

| **Variable** | **Correlation coefficient (r)** | **Magnitude** | **Relevance to covariate adjustment** |
| --- | --- | --- | --- |
| TG | 0.606 | Strong | Component of CTI; not included as a covariate |
| FPG | 0.457 | Moderate | Component of CTI; not included as a covariate |
| BMI | 0.413 | Moderate | Metabolically related variable; evaluated in sensitivity analysis |
| CRP | 0.405 | Moderate | Component of CTI; not included as a covariate |
| TC | 0.283 | Weak | Evaluated in sensitivity analysis |
| Age | 0.249 | Weak | Included in the primary model |
| eGFR | −0.187 | Weak | Alternative renal-function measure evaluated in sensitivity analysis |
| HGB | 0.056 | Negligible | Included in the extended clinical adjustment model |
| Serum creatinine | 0.045 | Negligible | Excluded from the primary model |

Correlations were assessed using Pearson correlation coefficients. Correlation magnitude was used to describe statistical association only and was not used to infer mediation or determine confounder status. CRP, TG, and FPG are direct components of the CTI calculation. Covariate selection for the primary model was based on the prespecified causal structure represented in the directed acyclic graph rather than on correlation coefficients alone.

**Supplementary Table S5. Alternative covariate specification sensitivity analyses**

**A. All-cause mortality**

| **Model** | **Q4 vs Q1, HR (95% CI)** | **P value** |
| --- | --- | --- |
| DAG-informed primary model | 1.54 (1.29–1.84) | <0.001 |
| Extended clinical adjustment model | 1.41 (1.20–1.67) | <0.001 |
| Extended clinical model + BMI | 1.46 (1.22–1.77) | <0.001 |
| Extended clinical model + diabetes and hyperlipidemia | 1.38 (1.15–1.66) | <0.001 |
| Extended clinical model + total cholesterol | 1.44 (1.22–1.71) | <0.001 |
| Extended clinical model with eGFR replacing CKD | 1.48 (1.25–1.75) | <0.001 |
| Original fully adjusted model* | 1.47 (1.19–1.80) | <0.001 |

Abbreviations: BMI, body mass index; CI, confidence interval; CKD, chronic kidney disease; CTI, C-reactive protein–triglyceride–glucose index; DAG, directed acyclic graph; eGFR, estimated glomerular filtration rate; HR, hazard ratio.

The DAG-informed primary model was adjusted for age, sex, race/ethnicity, educational attainment, marital status, poverty-income ratio, smoking status, and alcohol consumption. The extended clinical adjustment model additionally included hypertension, CKD, and hemoglobin. To examine the influence of additional metabolically related or potentially overlapping variables, BMI, diabetes and hyperlipidemia, and total cholesterol were separately added to the extended clinical adjustment model. In the alternative renal-function specification, eGFR replaced CKD rather than being entered concurrently with CKD. CRP, triglycerides, and fasting plasma glucose were not entered separately because they are direct components of CTI. HRs compare the highest CTI quartile (Q4) with the lowest quartile (Q1).

*The original fully adjusted model is shown only for comparison with the initial analysis and was not considered the primary model in the revised analysis. It included age, sex, race/ethnicity, educational attainment, marital status, poverty-income ratio, smoking status, alcohol consumption, hypertension, diabetes, hyperlipidemia, CKD, BMI, eGFR, hemoglobin, total cholesterol, and serum creatinine.

**Supplementary Table S5. B. Cardiovascular mortality**

| **Model** | **Q4 vs Q1, HR (95% CI)** | **P value** |
| --- | --- | --- |
| DAG-informed primary model | 1.78 (1.27–2.49) | <0.001 |
| Extended clinical adjustment model | 1.55 (1.12–2.14) | 0.008 |
| Extended clinical model + BMI | 1.39 (0.97–1.99) | 0.076 |
| Extended clinical model + diabetes and hyperlipidemia | 1.39 (0.98–1.98) | 0.066 |
| Extended clinical model + total cholesterol | 1.58 (1.14–2.18) | 0.006 |
| Extended clinical model with eGFR replacing CKD | 1.64 (1.19–2.27) | 0.003 |
| Original fully adjusted model* | 1.29 (0.88–1.90) | 0.194 |

Abbreviations: BMI, body mass index; CI, confidence interval; CKD, chronic kidney disease; CTI, C-reactive protein–triglyceride–glucose index; DAG, directed acyclic graph; eGFR, estimated glomerular filtration rate; HR, hazard ratio.

The DAG-informed primary model was adjusted for age, sex, race/ethnicity, educational attainment, marital status, poverty-income ratio, smoking status, and alcohol consumption. The extended clinical adjustment model additionally included hypertension, CKD, and hemoglobin. To examine the influence of additional metabolically related or potentially overlapping variables, BMI, diabetes and hyperlipidemia, and total cholesterol were separately added to the extended clinical adjustment model. In the alternative renal-function specification, eGFR replaced CKD rather than being entered concurrently with CKD. CRP, triglycerides, and fasting plasma glucose were not entered separately because they are direct components of CTI. HRs compare the highest CTI quartile (Q4) with the lowest quartile (Q1).

*The original fully adjusted model is shown only for comparison with the initial analysis and was not considered the primary model in the revised analysis. It included age, sex, race/ethnicity, educational attainment, marital status, poverty-income ratio, smoking status, alcohol consumption, hypertension, diabetes, hyperlipidemia, CKD, BMI, eGFR, hemoglobin, total cholesterol, and serum creatinine.

**Supplementary Table S6. Proportional hazards diagnostics and sensitivity analyses for the associations of CTI with all-cause and cardiovascular mortality**

**Part A. Summary of proportional hazards diagnostics**

| **Model and outcome** | **Events** | **CTI χ²** | **P value** | **Global χ²** | **P value** | **Interpretation** |
| --- | --- | --- | --- | --- | --- | --- |
| Primary DAG-informed model: all-cause mortality | 2,328 | 2.003 | 0.572 | 33.134 | 0.0045 | No evidence of nonproportionality for CTI; nonproportionality detected for selected covariates |
| Primary DAG-informed model: CVD mortality | 619 | 0.555 | 0.907 | 12.604 | 0.633 | No evidence of nonproportionality for CTI or the overall model |
| Extended clinical adjustment model: all-cause mortality | 2,296 | 1.070 | 0.784 | 62.120 | <0.001 | No evidence of nonproportionality for CTI; nonproportionality detected for selected covariates |
| Extended clinical adjustment model: CVD mortality | 615 | 0.720 | 0.867 | 21.150 | 0.272 | No evidence of nonproportionality for CTI or the overall model |

**Abbreviations:** CTI, C-reactive protein–triglyceride–glucose index; CVD, cardiovascular disease; DAG, directed acyclic graph; PH, proportional hazards.

**Note:** The PH assumption was evaluated using tests based on scaled Schoenfeld residuals from conventional unweighted Cox proportional hazards models specified with the same covariates as the corresponding survey-weighted models. These unweighted models were used for diagnostic and sensitivity analyses because standard Schoenfeld residual diagnostics are not directly applicable to survey-weighted Cox models. Primary effect estimates were obtained from survey-weighted Cox regression. A P value <0.05 indicates evidence against the PH assumption

**Supplementary Table S6. Part B. Proportional hazards assumption for the primary DAG-informed all-cause mortality model**

**Model 2 (primary DAG-informed model): n = 10,518; all-cause deaths = 2,328**

| **Variable** | **χ²** | **df** | **P value** | **Assessment** |
| --- | --- | --- | --- | --- |
| CTI quartiles | 2.003 | 3 | 0.572 | No evidence of PH violation |
| Age | 2.650 | 1 | 0.104 | No evidence of PH violation |
| Sex | 7.281 | 1 | **0.007** | Evidence of nonproportionality |
| Race/ethnicity | 5.661 | 3 | 0.129 | No evidence of PH violation |
| Education | 0.058 | 1 | 0.810 | No evidence of PH violation |
| Marital status | 3.396 | 1 | 0.065 | No evidence of PH violation |
| Poverty-income ratio | 2.678 | 1 | 0.102 | No evidence of PH violation |
| Smoking status | 3.680 | 2 | 0.159 | No evidence of PH violation |
| Alcohol consumption | 4.636 | 2 | 0.099 | No evidence of PH violation |
| GLOBAL | 33.134 | 15 | 0.0045 | Evidence of overall nonproportionality |

**Abbreviations:** CTI, C-reactive protein–triglyceride–glucose index; DAG, directed acyclic graph; PH, proportional hazards.

**Note:** The PH assumption was evaluated using tests based on scaled Schoenfeld residuals from an unweighted Cox proportional hazards model specified with the same covariates as the corresponding survey-weighted primary DAG-informed model. A P value <0.05 indicates evidence against the PH assumption. No evidence of nonproportionality was observed for CTI quartiles (P = 0.572). Sex showed evidence of nonproportionality (P = 0.007), and the global test was significant (P = 0.0045). Follow-up diagnostics after stratification by sex suggested additional nonproportionality for marital status and poverty-income ratio; these departures were therefore addressed in a PH-corrected sensitivity analysis (Part D).

**Supplementary Table S6. Part C. Proportional hazards assumption for the primary DAG-informed cardiovascular mortality model**

**Model 2 (primary DAG-informed model): n = 10,518; CVD deaths = 619**

| **Variable** | **χ²** | **df** | **P value** | **Assessment** |
| --- | --- | --- | --- | --- |
| CTI quartiles | 0.555 | 3 | 0.907 | No evidence of PH violation |
| Age | 0.392 | 1 | 0.531 | No evidence of PH violation |
| Sex | 0.468 | 1 | 0.494 | No evidence of PH violation |
| Race/ethnicity | 0.489 | 3 | 0.921 | No evidence of PH violation |
| Education | 0.166 | 1 | 0.683 | No evidence of PH violation |
| Marital status | 2.826 | 1 | 0.093 | No evidence of PH violation |
| Poverty-income ratio | 3.505 | 1 | 0.061 | No evidence of PH violation |
| Smoking status | 0.405 | 2 | 0.817 | No evidence of PH violation |
| Alcohol consumption | 2.077 | 2 | 0.354 | No evidence of PH violation |
| GLOBAL | 12.604 | 15 | 0.633 | No evidence of overall nonproportionality |

**Abbreviations:** CTI, C-reactive protein–triglyceride–glucose index; CVD, cardiovascular disease; DAG, directed acyclic graph; PH, proportional hazards.

**Note:** The PH assumption was evaluated using tests based on scaled Schoenfeld residuals from an unweighted Cox proportional hazards model specified with the same covariates as the corresponding survey-weighted primary DAG-informed model. A P value <0.05 indicates evidence against the PH assumption. No evidence of nonproportionality was observed for CTI quartiles (P = 0.907), any individual covariate (all P > 0.05), or the global model (P = 0.633). Therefore, no additional time-varying specification was required for the CVD mortality model.

**Supplementary Table S6. Part D. Sensitivity analysis accounting for nonproportional hazards in the primary DAG-informed all-cause mortality**

| **Model** | **CTI comparison** | **HR (95% CI)** | **P value** |
| --- | --- | --- | --- |
| Conventional unweighted Cox model | Q4 vs Q1 | 1.37 (1.19–1.58) | <0.001 |
| PH-corrected Cox model* | Q4 vs Q1 | 1.36 (1.18–1.58) | <0.001 |

Abbreviations: CI, confidence interval; CTI, C-reactive protein–triglyceride–glucose index; HR, hazard ratio; PH, proportional hazards.

Note: Both models included 10,518 participants and 2,328 all-cause deaths and contained the same DAG-informed covariate set. The conventional unweighted Cox model included age, sex, race/ethnicity, educational attainment, marital status, poverty-income ratio, smoking status, and alcohol consumption.
*The PH-corrected model was stratified by sex and marital status and included a time-interaction term between poverty-income ratio and log(1 + follow-up time). The Q4-versus-Q1 estimate was essentially unchanged after accounting for nonproportional hazards, supporting the robustness of the CTI–all-cause mortality association. These unweighted models were used only as sensitivity analyses; the primary effect estimates were obtained from survey-weighted Cox regression.

**Supplementary Table S6. Part E. Proportional hazards assumption for the extended clinical adjustment model: all-cause mortality**

**Model 3 (extended clinical adjustment model): n = 10,415; all-cause deaths = 2,296**

| **Variable** | **χ²** | **df** | **P value** | **Assessment** |
| --- | --- | --- | --- | --- |
| CTI quartiles | 1.070 | 3 | 0.784 | No evidence of PH violation |
| Age | 7.858 | 1 | 0.005 | Evidence of nonproportionality |
| Sex | 5.414 | 1 | 0.020 | Evidence of nonproportionality |
| Race/ethnicity | 6.604 | 3 | 0.086 | No evidence of PH violation |
| Education | 0.133 | 1 | 0.716 | No evidence of PH violation |
| Marital status | 2.289 | 1 | 0.130 | No evidence of PH violation |
| Poverty-income ratio | 2.481 | 1 | 0.115 | No evidence of PH violation |
| Smoking status | 5.556 | 2 | 0.062 | No evidence of PH violation |
| Alcohol consumption | 6.153 | 2 | 0.046 | Evidence of nonproportionality |
| Hypertension | 0.630 | 1 | 0.428 | No evidence of PH violation |
| Chronic kidney disease | 0.352 | 1 | 0.553 | No evidence of PH violation |
| Hemoglobin | 13.061 | 1 | <0.001 | Evidence of nonproportionality |
| GLOBAL | 62.120 | 18 | <0.001 | Evidence of overall nonproportionality |

Abbreviations: CTI, C-reactive protein–triglyceride–glucose index; PH, proportional hazards.

Note: The PH assumption was evaluated using tests based on scaled Schoenfeld residuals from an unweighted Cox proportional hazards model specified with the same covariates as the corresponding survey-weighted extended clinical adjustment model. A P value <0.05 indicates evidence against the PH assumption. No evidence of nonproportionality was observed for CTI quartiles (P = 0.784). The global test was significant (P < 0.001), with evidence of nonproportionality for age, sex, alcohol consumption, and hemoglobin.

**Supplementary Table S6. Part F. Proportional hazards assumption for the extended clinical adjustment model: cardiovascular mortality**

**Model 3 (extended clinical adjustment model): n = 10,415; CVD deaths = 615**

| **Variable** | **χ²** | **df** | **P value** | **Assessment** |
| --- | --- | --- | --- | --- |
| CTI quartiles | 0.720 | 3 | 0.867 | No evidence of PH violation |
| Age | 1.890 | 1 | 0.170 | No evidence of PH violation |
| Sex | 0.150 | 1 | 0.698 | No evidence of PH violation |
| Race/ethnicity | 0.810 | 3 | 0.846 | No evidence of PH violation |
| Education | 0.330 | 1 | 0.563 | No evidence of PH violation |
| Marital status | 1.980 | 1 | 0.160 | No evidence of PH violation |
| Poverty-income ratio | 2.940 | 1 | 0.086 | No evidence of PH violation |
| Smoking status | 0.820 | 2 | 0.665 | No evidence of PH violation |
| Alcohol consumption | 2.150 | 2 | 0.341 | No evidence of PH violation |
| Hypertension | 2.830 | 1 | 0.092 | No evidence of PH violation |
| Chronic kidney disease | 0.780 | 1 | 0.376 | No evidence of PH violation |
| Hemoglobin | 3.350 | 1 | 0.067 | No evidence of PH violation |
| GLOBAL | 21.150 | 18 | 0.272 | No evidence of overall nonproportionality |

Abbreviations: CTI, C-reactive protein–triglyceride–glucose index; CVD, cardiovascular disease; PH, proportional hazards.

Note: The PH assumption was evaluated using tests based on scaled Schoenfeld residuals from an unweighted Cox proportional hazards model specified with the same covariates as the corresponding survey-weighted extended clinical adjustment model. A P value <0.05 indicates evidence against the PH assumption. No evidence of nonproportionality was observed for CTI quartiles (P = 0.867), and the global test was not significant (P = 0.272), providing no evidence of overall nonproportionality in the extended CVD mortality model.

**Supplementary Table S6. Part G. Sensitivity analysis accounting for nonproportional hazards in the extended clinical all-cause mortality model**

| **Model** | **CTI comparison** | **HR (95% CI)** | **P value** |
| --- | --- | --- | --- |
| Conventional unweighted Cox model | Q4 vs Q1 | 1.28 (1.11–1.49) | <0.001 |
| PH-corrected Cox model | Q4 vs Q1 | 1.27 (1.10–1.47) | 0.001 |

Abbreviations: CI, confidence interval; CTI, C-reactive protein–triglyceride–glucose index; HR, hazard ratio; PH, proportional hazards.

Note: Both models included 10,415 participants and 2,296 all-cause deaths and contained the covariates of the extended clinical adjustment model.
*The PH-corrected model was stratified by sex and alcohol consumption and included time-interaction terms between age and log(1 + follow-up time) and between hemoglobin and log(1 + follow-up time). The Q4-versus-Q1 estimate changed only minimally after accounting for nonproportional hazards, supporting the robustness of the CTI association in the extended clinical model. These unweighted models were used only as sensitivity analyses; the primary effect estimates were obtained from survey-weighted Cox regression.

**Supplementary Table S7. Survey-weighted Fine–Gray competing-risk analyses of the association between CTI and cardiovascular mortality**

| **Model** | **CTI specification** | **sHR (95% CI)** | **P value** |
| --- | --- | --- | --- |
| Primary DAG-informed model | CTI per 1-unit increase | 1.35 (1.20–1.53) | <0.001 |
|  | Q1 | Reference | — |
|  | Q2 vs Q1 | 1.25 (0.85–1.82) | 0.257 |
|  | Q3 vs Q1 | 1.46 (0.99–2.14) | 0.055 |
|  | Q4 vs Q1 | 1.82 (1.28–2.60) | <0.001 |
| Extended clinical adjustment model | CTI per 1-unit increase | 1.28 (1.14–1.45) | <0.001 |
|  | Q1 | Reference | — |
|  | Q2 vs Q1 | 1.19 (0.83–1.73) | 0.346 |
|  | Q3 vs Q1 | 1.37 (0.96–1.97) | 0.087 |
|  | Q4 vs Q1 | 1.62 (1.15–2.28) | 0.006 |

Abbreviations: CI, confidence interval; CTI, C-reactive protein–triglyceride–glucose index; CVD, cardiovascular disease; DAG, directed acyclic graph; sHR, subdistribution hazard ratio.

Non-CVD death was treated as the competing event for cardiovascular mortality. Fine–Gray expanded data were generated using a counting-process representation. Fine–Gray interval weights were combined with the NHANES fasting-subsample sampling weights, and the original NHANES strata and primary sampling units were retained in the survey design. Survey-weighted Cox regression was then fitted to the Fine–Gray expanded data to estimate subdistribution hazard ratios (sHRs) and corresponding 95% confidence intervals. The primary DAG-informed model was adjusted for age, sex, race/ethnicity, educational attainment, marital status, poverty-income ratio, smoking status, and alcohol consumption. The extended clinical adjustment model additionally included hypertension, chronic kidney disease, and hemoglobin. Q1 served as the reference category in quartile analyses.

**Supplementary Figure S1. Directed acyclic graph (DAG) illustrating the hypothesized causal structure for the association between the C-reactive protein–triglyceride–glucose index (CTI) and mortality.**

Supplementary Figure S1. Directed acyclic graph (DAG) illustrating the hypothesized causal structure for the association between the C-reactive protein–triglyceride–glucose index (CTI) and mortality. Under the causal assumptions represented in the DAG, the minimally sufficient adjustment set comprised age, sex, race/ethnicity, educational attainment, marital status, poverty-income ratio, smoking status, and alcohol consumption. CRP, triglycerides, and fasting plasma glucose were not considered separate adjustment covariates because they are direct components of CTI. Additional metabolic, clinical, and renal variables were evaluated in extended or sensitivity models because of their potential downstream roles or substantial information overlap.

**Supplementary Figure S2. Scaled Schoenfeld residual plots for the primary DAG-informed Cox model of all-cause mortality.**

Scaled Schoenfeld residuals were examined using a conventional unweighted Cox proportional hazards model specified with the same covariates as the corresponding survey-weighted primary DAG-informed model. The diagnostic model included 10,518 participants and 2,328 all-cause deaths. There was no evidence of nonproportionality for CTI quartiles (P = 0.572). Sex showed evidence of nonproportionality (P = 0.007), whereas the remaining individual covariates did not show statistically significant violations in the initial diagnostic model. The global test was significant (P = 0.0045). DAG, directed acyclic graph; CTI, C-reactive protein–triglyceride–glucose index.

**Supplementary Figure S3. Scaled Schoenfeld residual plots for the primary DAG-informed Cox model of cardiovascular mortality.**

Scaled Schoenfeld residuals were examined using a conventional unweighted Cox proportional hazards model specified with the same covariates as the corresponding survey-weighted primary DAG-informed model. The diagnostic model included 10,518 participants and 619 cardiovascular deaths. There was no evidence of nonproportionality for CTI quartiles (P = 0.907), any individual covariate (all P > 0.05), or the global model (P = 0.633). DAG, directed acyclic graph; CTI, C-reactive protein–triglyceride–glucose index.

**Supplementary Figure S4. Scaled Schoenfeld residual plots for the extended clinical adjustment Cox model of all-cause mortality.**

Supplementary Figure S4. Scaled Schoenfeld residual plots for the extended clinical adjustment Cox model of all-cause mortality. Scaled Schoenfeld residuals were examined using a conventional unweighted Cox proportional hazards model specified with the same covariates as the corresponding survey-weighted extended clinical adjustment model (Model 3). The diagnostic model included 10,415 participants and 2,296 all-cause deaths. There was no evidence of nonproportionality for CTI quartiles (P = 0.784). Evidence of nonproportionality was observed for age (P = 0.005), sex (P = 0.020), alcohol consumption (P = 0.046), and hemoglobin (P < 0.001), and the global test was significant (P < 0.001). CTI, C-reactive protein–triglyceride–glucose index.

**Supplementary Figure S5. Scaled Schoenfeld residual plots for the extended clinical adjustment Cox model of cardiovascular mortality.**

Supplementary Figure S5. Scaled Schoenfeld residual plots for the extended clinical adjustment Cox model of cardiovascular mortality. Scaled Schoenfeld residuals were examined using a conventional unweighted Cox proportional hazards model specified with the same covariates as the corresponding survey-weighted extended clinical adjustment model (Model 3). The diagnostic model included 10,415 participants and 615 cardiovascular deaths. There was no evidence of nonproportionality for CTI quartiles (P = 0.867), any individual covariate (all P > 0.05), or the global model (P = 0.272). CTI, C-reactive protein–triglyceride–glucose index.

**Supplementary Figure S6.** Calibration of the prediction-oriented base model and the base model plus CTI for all-cause and cardiovascular mortality.

The prediction-oriented base model included age, sex, race/ethnicity, smoking status, hypertension, diabetes mellitus, body mass index, total cholesterol, HDL cholesterol, and chronic kidney disease; the augmented model additionally included continuous CTI. Both models were fitted in the same complete-case prediction sample (n = 10,279). Panels A–C show calibration for all-cause mortality at 5, 10, and 15 years, respectively, and panels D–F show calibration for cardiovascular mortality at 5, 10, and 15 years, respectively. Calibration was evaluated in deciles of predicted risk. Observed all-cause mortality risk was estimated using local Kaplan–Meier methods, whereas observed cardiovascular risk was estimated using local Aalen–Johansen methods with non-cardiovascular death treated as a competing event. The diagonal reference line represents perfect agreement between predicted and observed risks. These curves represent apparent calibration in the model-development sample; no cross-validation or optimism-corrected internal validation was applied. CTI, C-reactive protein–triglyceride–glucose index; CVD, cardiovascular disease.

**Supplementary Figure S7.** Decision curve analysis of the prediction-oriented base model and the base model plus CTI for all-cause and cardiovascular mortality.

The prediction-oriented base model included age, sex, race/ethnicity, smoking status, hypertension, diabetes mellitus, body mass index, total cholesterol, HDL cholesterol, and chronic kidney disease; the augmented model additionally included continuous CTI. Both models were fitted in the same complete-case prediction sample (n = 10,279). Panels A–C show decision curve analyses for all-cause mortality at 5, 10, and 15 years, respectively, and panels D–F show the corresponding analyses for cardiovascular mortality at 5, 10, and 15 years, respectively. Net benefit was evaluated across the displayed threshold probabilities using model-predicted risk at each time horizon. The treat-all and treat-none strategies are shown as reference strategies. For cardiovascular mortality, non-cardiovascular death was treated as a competing event. The curves represent apparent performance from the model-development sample and were not internally validated by cross-validation or optimism correction. CTI, C-reactive protein–triglyceride–glucose index; CVD, cardiovascular disease.
